# Supplementary material for: Introgression and disruption of migration routes have shaped the genetic integrity of wildebeest populations
Source: Nat Commun. 2024 Apr 12;15:2921. doi: 10.1038/s41467-024-47015-y (PMC11014984; doi:10.1038/s41467-024-47015-y)
Supplement: Supplementary file 1 — Supplementary Information [file 41467_2024_47015_MOESM1_ESM.pdf]

Introgression and disruption of migration routes have shaped the genetic integrity of wildebeest populations

AUTHOR LIST

AUTHOR LIST

Xiaodong Liu[1#], Long Lin[1#], Mikkel-Holger S. Sinding[1#], Laura D. Bertola[1], Kristian Hanghøj[1], Liam Quinn[1], Genís Garcia-Erill[1], Malthe Sebro Rasmussen[1], Mikkel Schubert[2], Patrícia Pečnerová[1], Renzo F. Balboa[1], Zilong Li[1], Michael P. Heaton[3], Timothy P. L. Smith[3,], Rui Resende-Pinto[4,5], Xi Wang[1], Josiah Kuja[1], Anna Brüniche-Olsen[1], Jonas Meisner[2,6], Cindy G. Santander[1], Joseph O. Ogutu[7], Charles Masembe[8], Rute R. da Fonseca[4,5], Vincent Muwanika[9], Hans R. Siegismund[1], Anders Albrechtsen[1]*, Ida Moltke[1]*, Rasmus Heller[1]*

[#] These authors contributed equally

* These authors jointly supervised this work

AFFILIATIONS

[1] Department of Biology, University of Copenhagen, Copenhagen, Denmark

[2] Novo Nordisk Foundation Center for Basic Metabolic Research, University of Copenhagen, Copenhagen, Denmark

[3] USDA, ARS, U.S. Meat Animal Research Center (USMARC), Clay Center, Nebraska, USA

[4] CIIMAR – Interdisciplinary Centre of Marine and Environmental Research – University of Porto, Porto, Portugal

[5] Section for Biodiversity, Globe Institute, University of Copenhagen, Copenhagen, Denmark

[6] Copenhagen Research Centre for Mental Health, Copenhagen University Hospital, Copenhagen, Denmark

[7] Biostatistics Unit, Institute of Crop Science, University of Hohenheim, Stuttgart, Germany

[8] Department of Zoology, Entomology and Fisheries Sciences, Makerere University, P. O. Box 7062, Kampala, Uganda

[9] Department of Environmental Management, Makerere University, PO Box 7062, Kampala, Uganda

Supplementary Information

Supplementary Figures **5**

Supplementary Figure 1. Neighbor-joining tree for 131 wildebeest samples based on identity-by-state (IBS) distances.. 5

Supplementary Figure 2. Admixture proportions of the blue wildebeest.. 6

Supplementary Figure 3. Evaluations of the ADMIXTURE models based on the blue wildebeest. 7

Supplementary Figure 4. Admixture proportions of the brindled wildebeest.. 9

Supplementary Figure 5. Evaluations of the ADMIXTURE models based on the brindled wildebeest.. 10

Supplementary Figure 6. Patterns of linkage disequilibrium (LD) decay for all wildebeest populations with at least 5 samples. 11

Supplementary Figure 7. Admixture proportions of the black wildebeest.. 12

Supplementary Figure 8. Evaluations of the ADMIXTURE models based on the black wildebeest. 13

Supplementary Figure 9. Admixture proportions of the black and blue wildebeest for all 131 samples.. 14

Supplementary Figure 10. Evaluations of the ADMIXTURE models based on the black and blue wildebeest. 15

Supplementary Figure 11. Boxplot of the Z-scores from D-statistics of the black wildebeest population in Namibia.. 16

Supplementary Figure 12. Heterozygosity and ROH proportion for wildebeest populations.. 17

Supplementary Figure 13. Statistically non-rejected admixture graphs with the number of admixture events ranging from 0 to 5 in qpGraph.. 18

Supplementary Figure 14. Frequency spectrum of D-statistics (DFS) between the blue wildebeest populations and the black wildebeest. 20

Supplementary Figure 15. Phylogenetic tree using mitochondrial DNA including wildebeest and outgroups. 21

Supplementary Figure 16. Local ancestries inferred using LOTER in all samples of the brindled population in Etosha (B-Etosha) excluding the one shown in Fig. 3B. 22

Supplementary Figure 17. Local ancestries inferred using LOTER in all samples of the Nyassa population excluding the one shown in Fig. 3B. 23

Supplementary Figure 18. Likelihoods and parameter point estimates of the fastsimcoal2 demographic models. 24

Supplementary Figure 19. Genomic landscapes of differentiation and nucleotide diversity in wildebeest. 25

Supplementary Figure 20. Characterization of the highly differentiated region on chromosome 1. 26

Supplementary Figure 21. Alignment between chromosome 1 of wildebeest and chromosomes 2, 25 of domestic goat. 27

Supplementary Figure 22. Genotype calls, proportions of heterozygous sites and SNP density for ROH validation in a black wildebeest sample (CGnoNaC_2630). 28

Supplementary Figure 23. Genotype calls, proportions of heterozygous sites and SNP density for ROH validation in a Brindled wildebeest sample (CTauBwC__761). 29

Supplementary Figure 24. Genotype calls, proportions of heterozygous sites and SNP density for

ROH validation in a Cookson wildebeest sample (CTauZmE_2542). ... 30
Supplementary Figure 25. Genotype calls, proportions of heterozygous sites and SNP density for ROH validation in a Nyassa wildebeest sample (CTauTzS_3711). ... 31
Supplementary Figure 26. Genotype calls, proportions of heterozygous sites and SNP density for ROH validation in an East white-bearded wildebeest sample (CTauKeS__716). ... 32
Supplementary Note ... 34
Supplementary Note 1. Population homogeneity criteria. ... 34
Supplementary Note 2. Historical records and identification of migratory and non-migratory populations in eastern and southern Africa.. ... 36
Supplementary References ... 39

Supplementary Figures

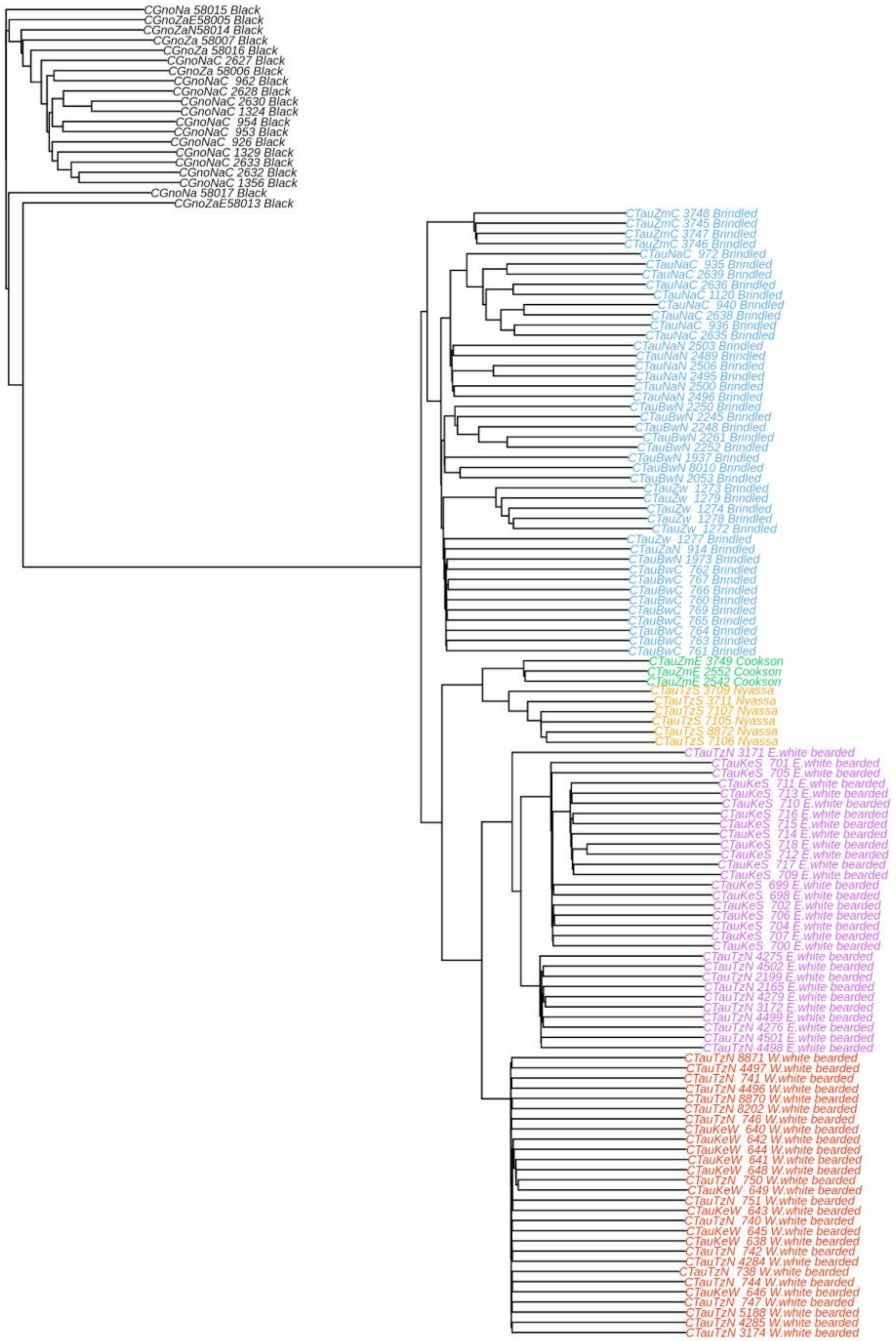

Supplementary Figure 1. Neighbor-joining tree for 131 wildebeest samples based on identity-by-state (IBS) distances. Colors represent different species/subspecies. IBS distances were estimated by PLINK based on the imputed SNPs (dataset1).

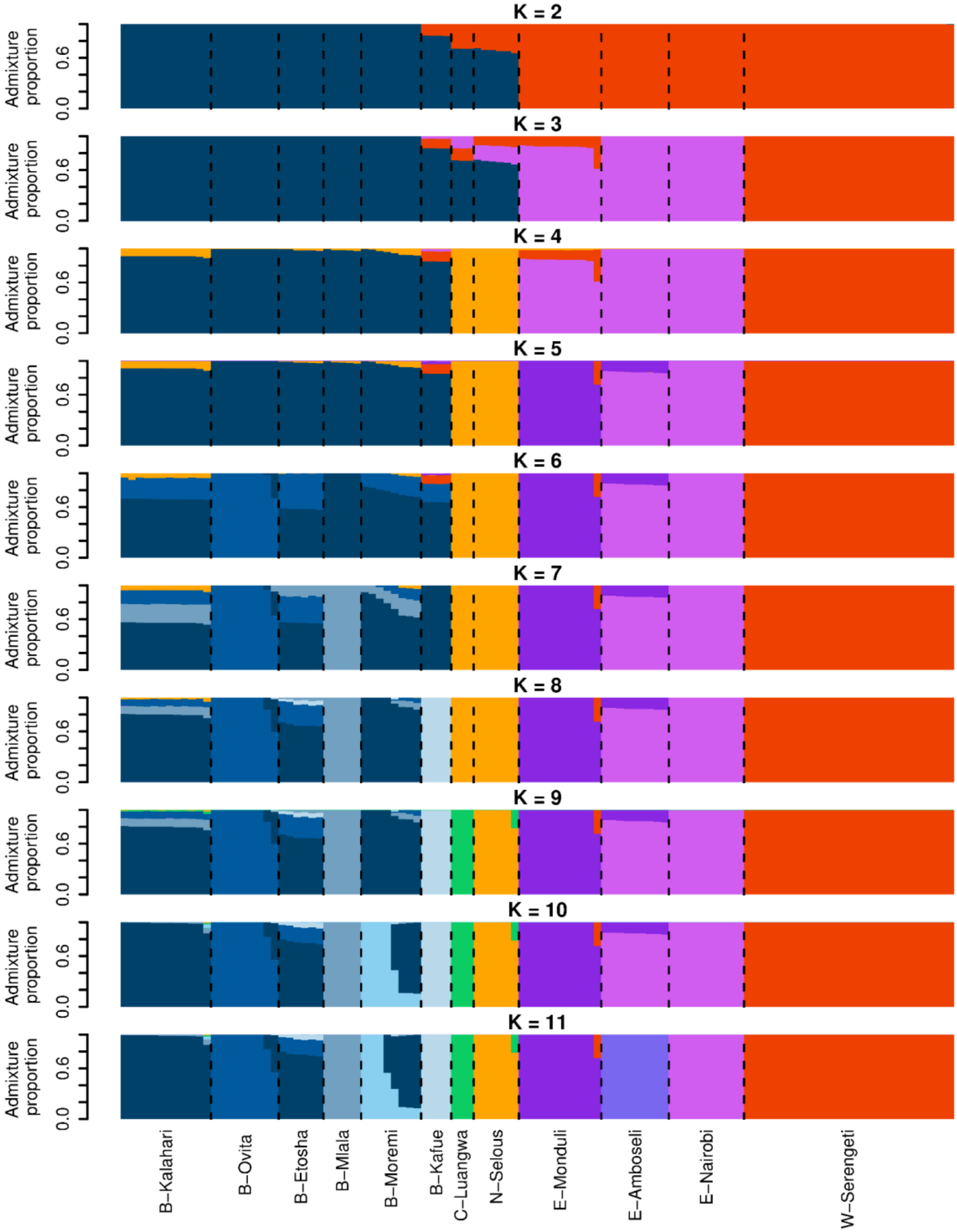

Supplementary Figure 2. Admixture proportions of the blue wildebeest. Ancestry proportions of the blue wildebeest were inferred by ADMIXTURE with the assumed number of ancestry (K) ranging from 2 to 11. First degree relatives and duplicates were excluded prior to the analysis. Sites with minor allele frequency (MAF) lower than 0.05 and high linkage disequilibrium (LD, $r^2 > 0.7$) were omitted from the analysis. ADMIXTURE analysis was conducted based on 0.9 million SNPs, which were randomly selected from the filtered sites.

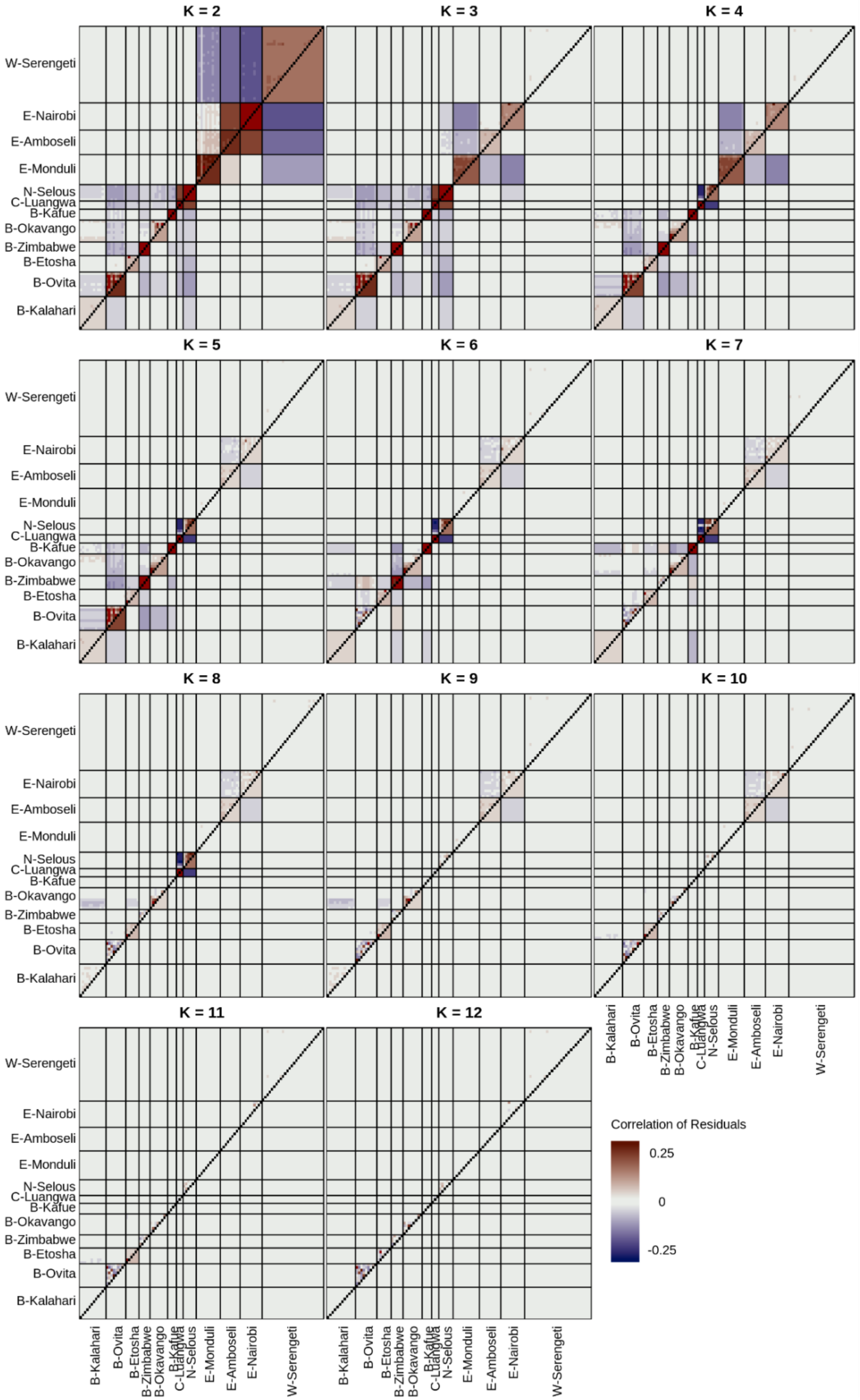
K = 2
K = 3
K = 4
K = 5
K = 6
K = 7
K = 8
K = 9
K = 10
K = 11
K = 12
W-Serengeti
E-Nairobi
E-Amboseli
E-Monduli
N-Selous
C-Luangwa
B-Kafue
B-Okavango
B-Zimbabwe
B-Etosha
B-Ovita
B-Kalahari
Correlation of Residuals
0.25
0
-0.25

Supplementary Figure 3. Evaluations of the ADMIXTURE models based on the blue wildebeest. Correlations of residuals of the ADMIXTURE results for blue wildebeest with K ranging from 2 to 12 (Figs 1 and S2) were estimated using evalAdmix. In each panel, pairwise correlations of residuals between individuals are shown above the diagonal, while average correlations of residuals for all individual pairs within populations are shown below the diagonal. Samples were placed in the same order as the ADMIXTURE plot.

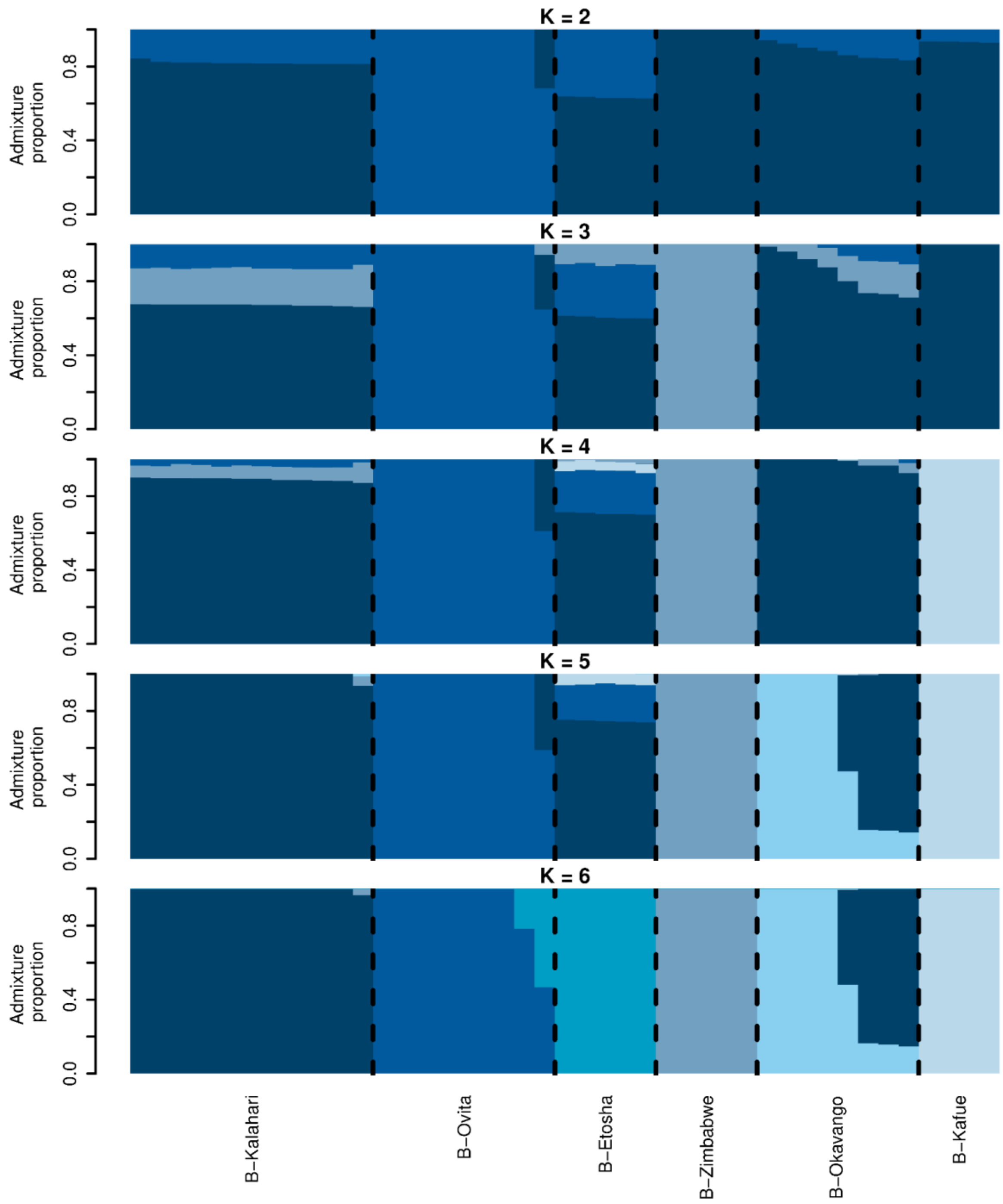

Supplementary Figure 4. Admixture proportions of the brindled wildebeest. Ancestry proportions of the blue wildebeest were inferred by ADMIXTURE with the assumed number of ancestry (K) ranging from 2 to 6. One sample of a second degree related pair in B-Etosha was excluded prior to the analysis. The analysis was based on the imputed SNPs (dataset1). Individuals were ordered by their estimated ancestry proportions at each K.

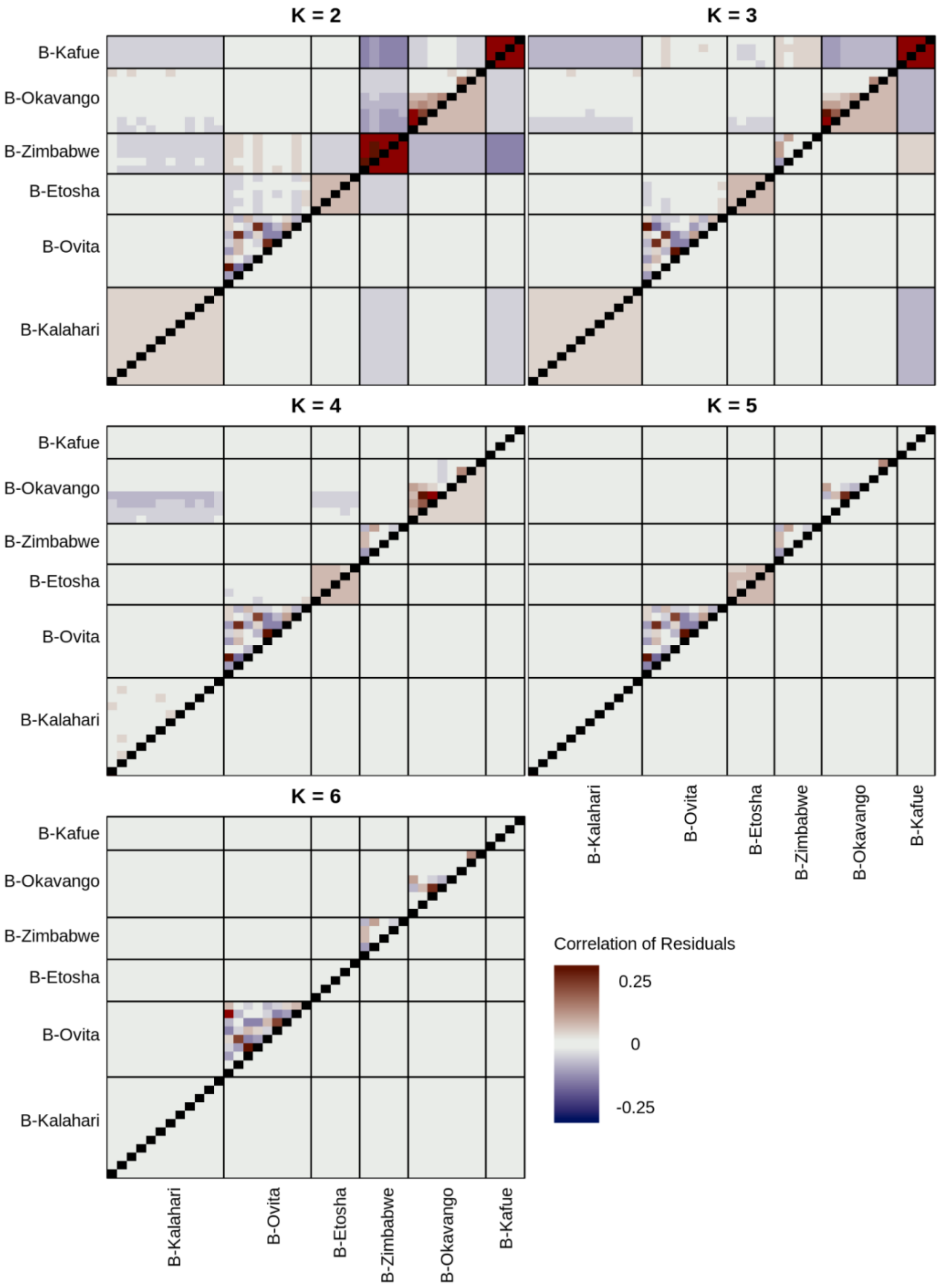

Supplementary Figure 5. Evaluations of the ADMIXTURE models based on the brindled wildebeest. Correlations of residuals of the ADMIXTURE results for brindled wildebeest with K ranging from 2 to 6 (Figs 1 and S2) were estimated using evalAdmix. In each panel, pairwise correlations of residuals between individuals are shown above the diagonal, while average correlations of residuals for all individual pairs within populations are shown below the diagonal. Samples were placed in the same order as the ADMIXTURE plot.

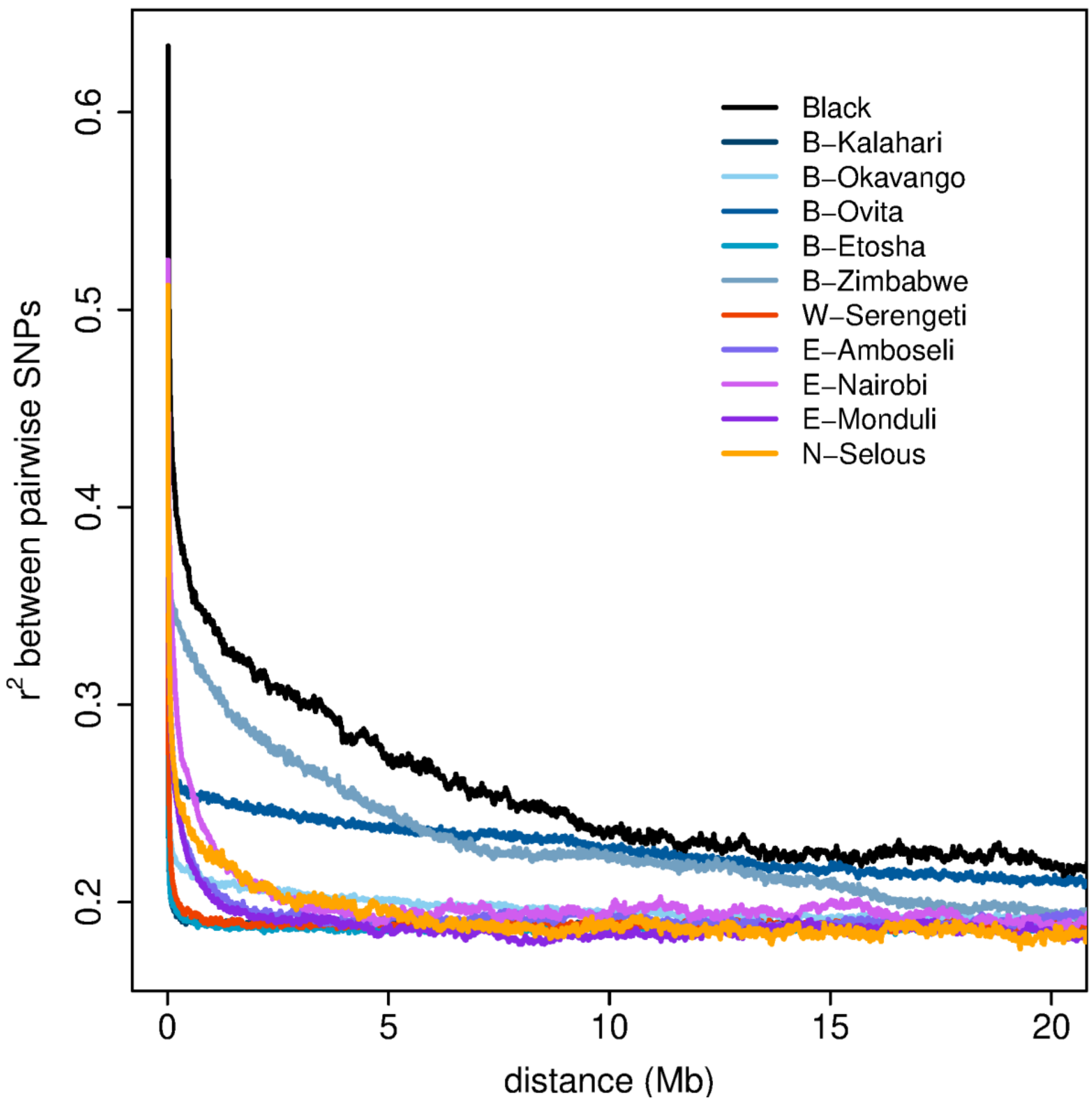

Supplementary Figure 6. Patterns of linkage disequilibrium (LD) decay for all wildebeest populations with at least 5 samples. For each considered population, LD was measured as mean r^2 for SNP pairs stratified by the genomic distances.To make the curves comparable, all populations were downsampled to 5 individuals.

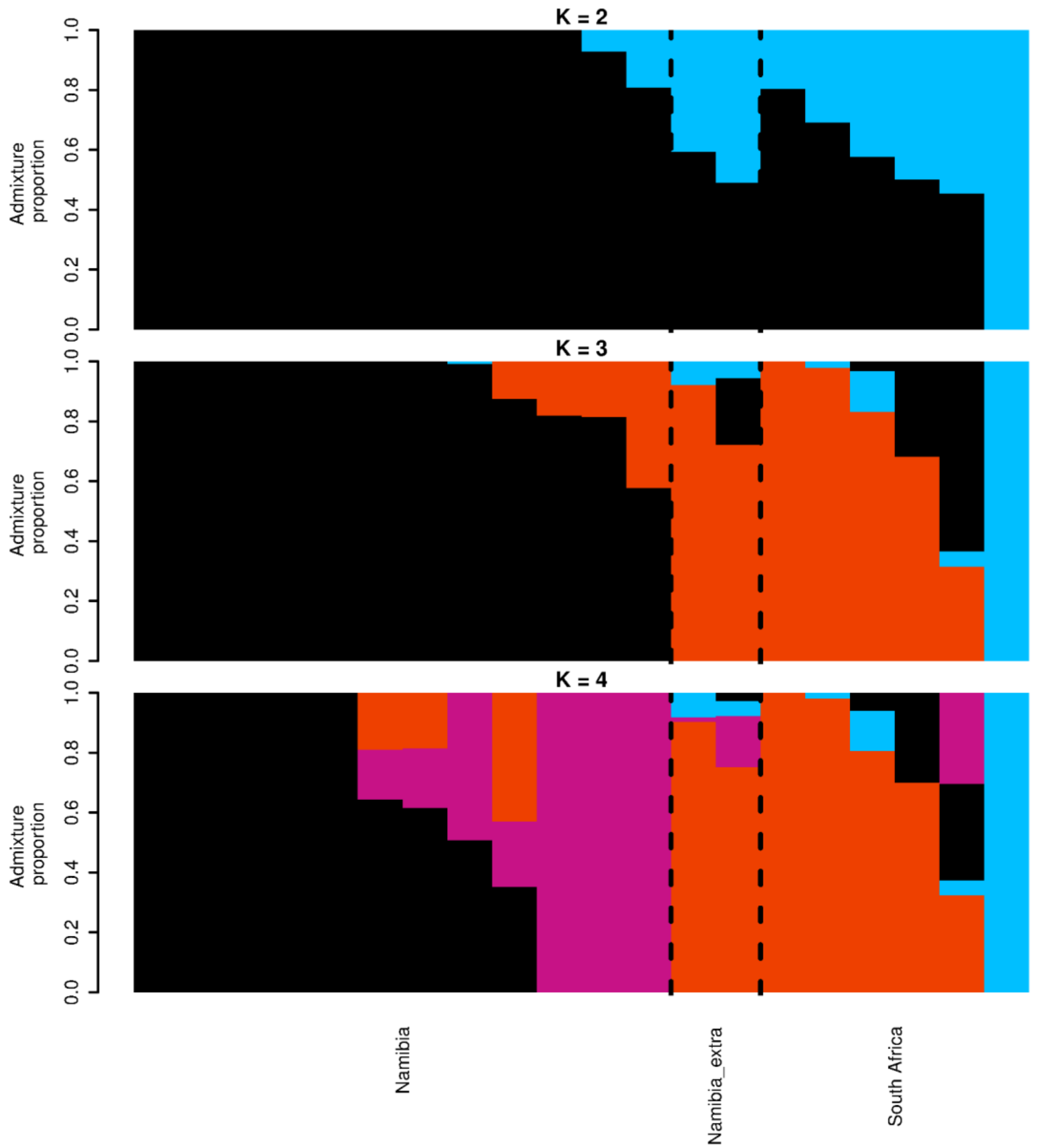

Supplementary Figure 7. Admixture proportions of the black wildebeest. Ancestry proportions of the black wildebeest were inferred by the ADMIXTURE with assumed number of ancestry (K) ranging from 2 to 4. We extracted the samples from the imputed dataset and filtered out sites with MAF lower than 0.05, missing call frequencies greater than 0.05, or in high LD ($r^2 > 0.8$) before running ADMIXTURE. Individuals were ordered by their estimated ancestry proportions at each K.

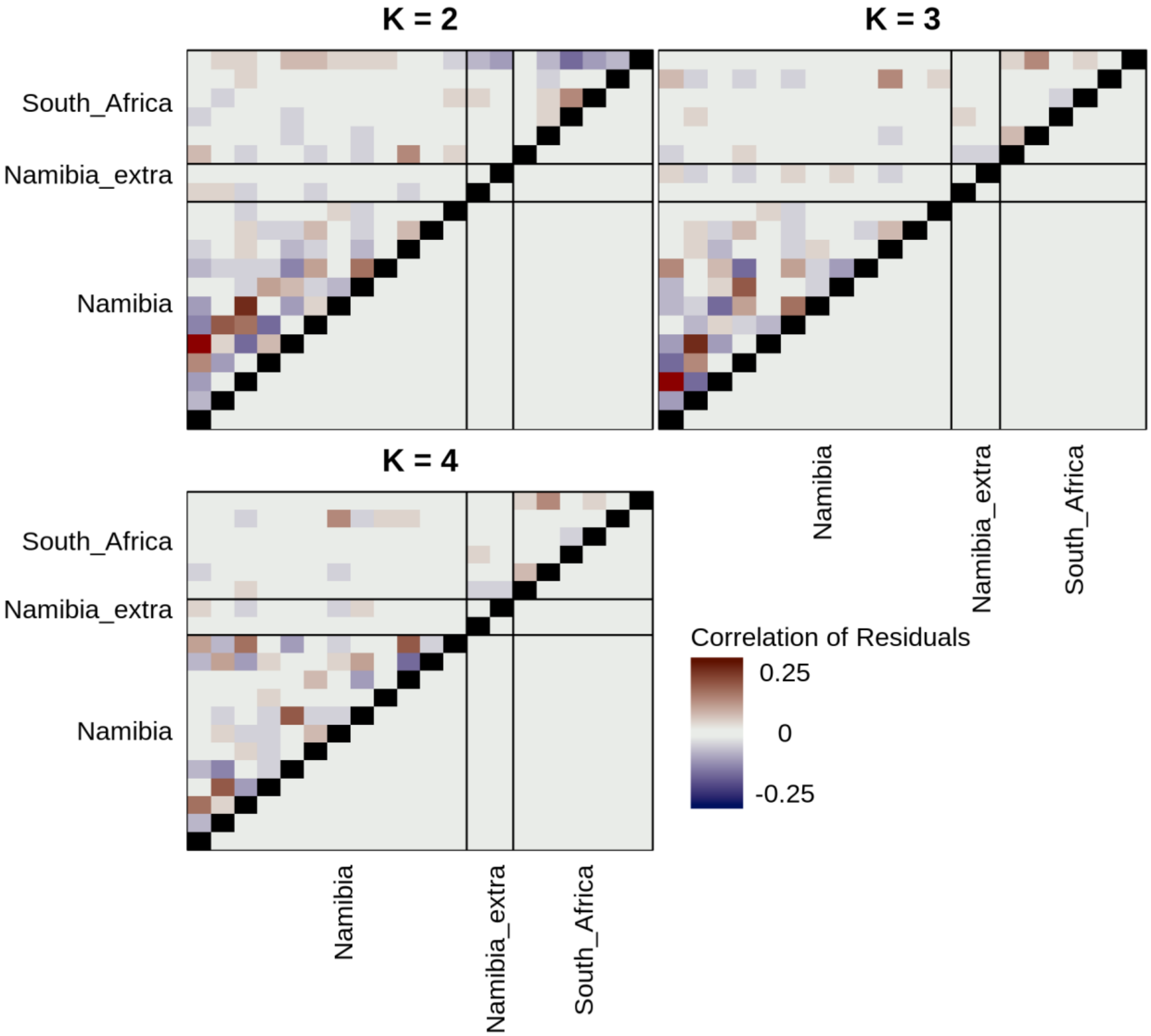

Supplementary Figure 8. Evaluations of the ADMIXTURE models based on the black wildebeest. Correlations of residuals of the ADMIXTURE results for brindled wildebeest with K ranging from 2 to 4 (Figs 1 and S2) were estimated using evalAdmix. In each panel, pairwise correlations of residuals between individuals are shown above the diagonal, while average correlations of residuals for all individual pairs within populations are shown below the diagonal. Samples were placed in the same order as the ADMIXTURE plot.

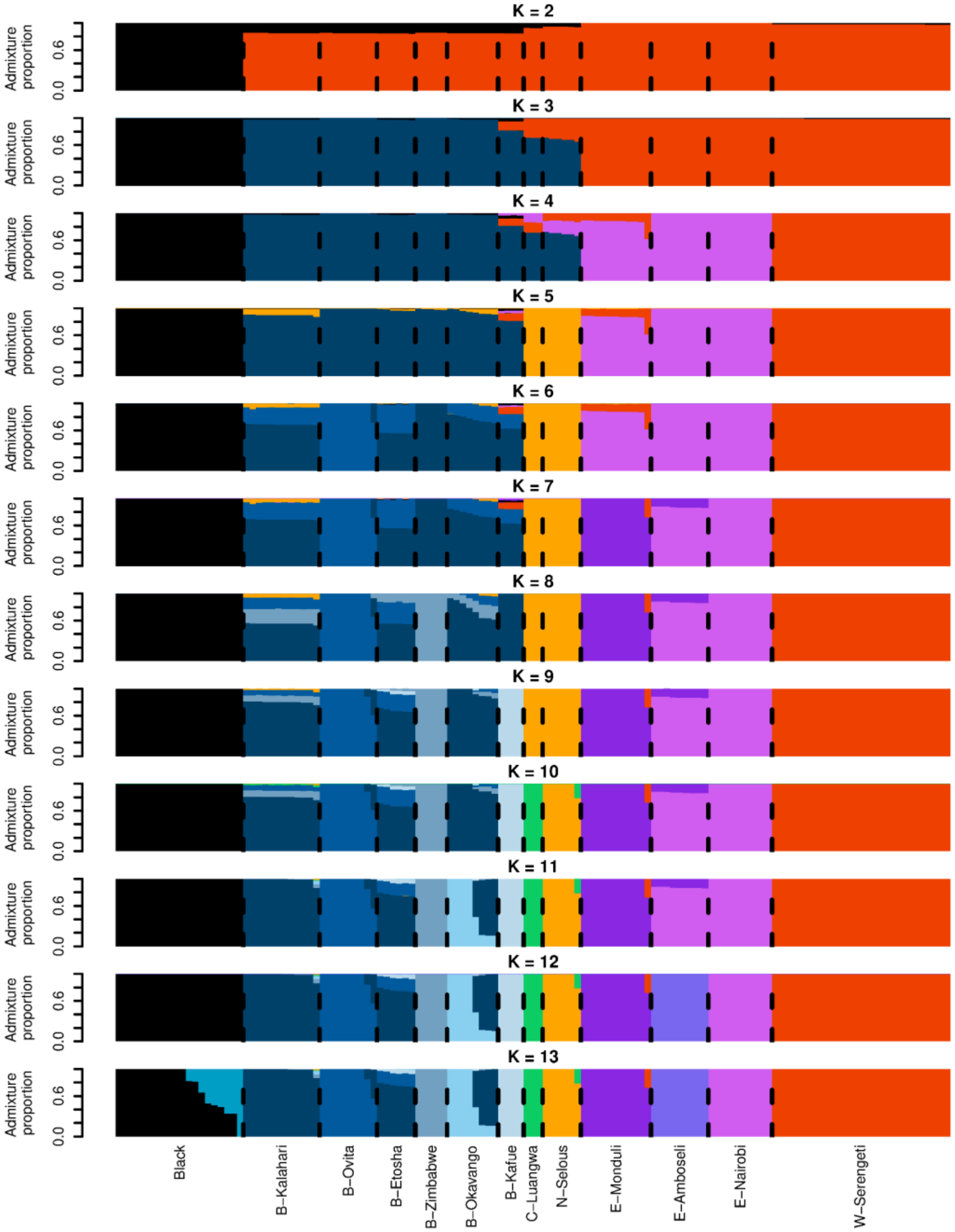

Supplementary Figure 9. Admixture proportions of the black and blue wildebeest for all 131 samples. Ancestry proportions of the black and blue wildebeest were inferred by the ADMIXTURE with assumed number of ancestry (K) ranging from 2 to 13. We used the imputed dataset and filtered out sites with high LD ($r^2 > 0.6$). We then randomly selected one million SNPs for the ADMIXTURE analysis. Individuals were ordered by their estimated ancestry proportions at each K.

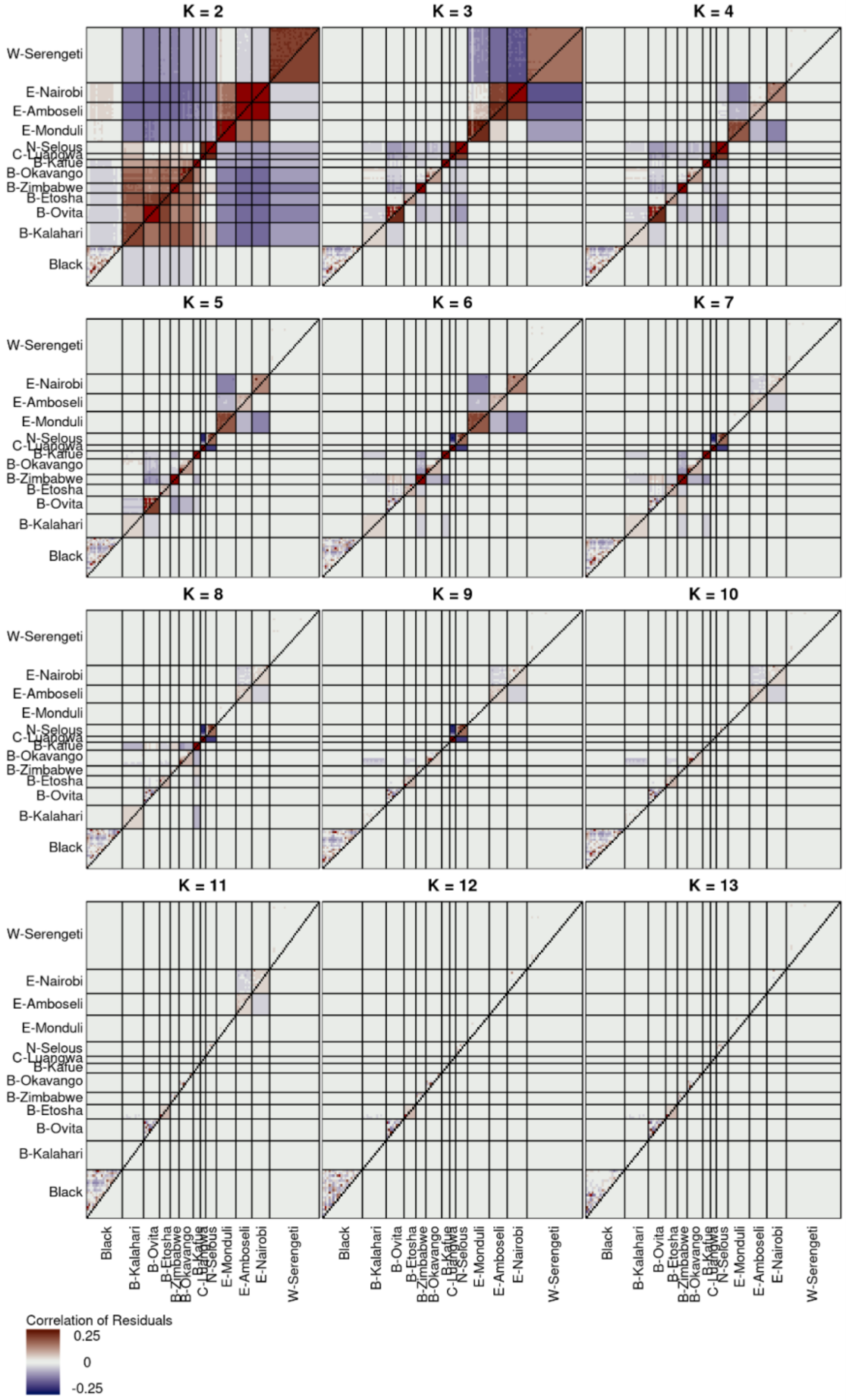

Supplementary Figure 10. Evaluations of the ADMIXTURE models based on the black and blue wildebeest.

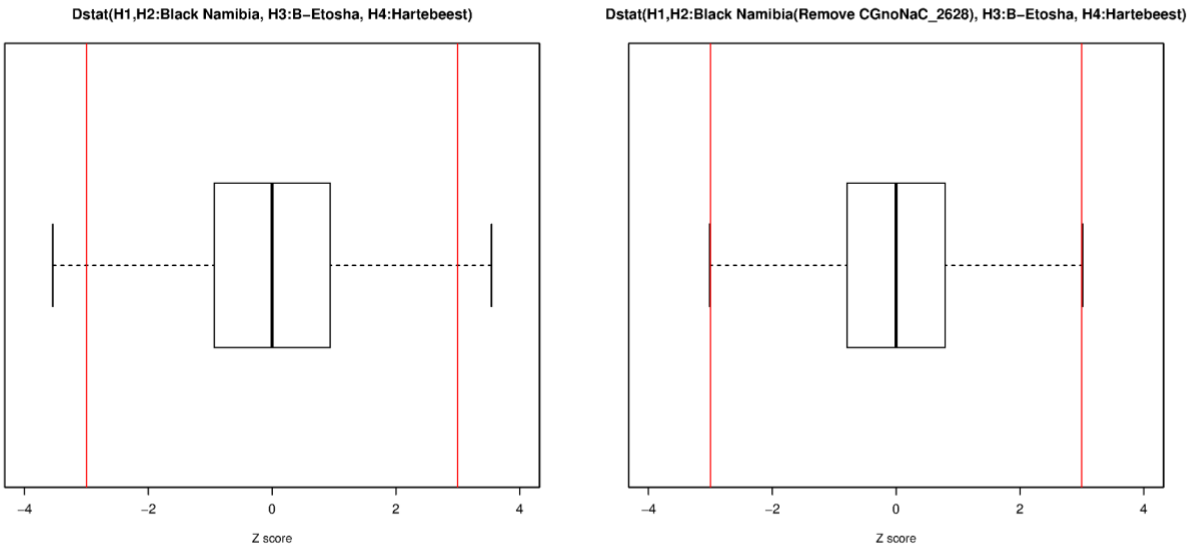

Supplementary Figure 11. Boxplot of the Z-scores from *D*-statistics of the black wildebeest population in Namibia. Ten black wildebeests from Namibia, which were estimated to be non-admixed at K=2 (Fig. S7) were selected for genetic homogeneity test using *D*-statistics. Pairwise black wildebeest individuals were used as H1 and H2, with B-Etosha as H3 and hartebeest as H4 (outgroup). *D*-statistics were calculated by the 'qpdstat' function implemented in the R package admixtools2. Z scores larger than 3 or smaller than -3 (red lines in the plot) are deemed to be significant. All pairs showing significant D-statistics in the left panel were caused by a single individual-CGnoNaC_2628. After removing the individual, no pairs showed significant D-statistics (right panel). Thus, CGnoNaC_2628 was excluded from the subset of the homogenous population of black wildebeest.

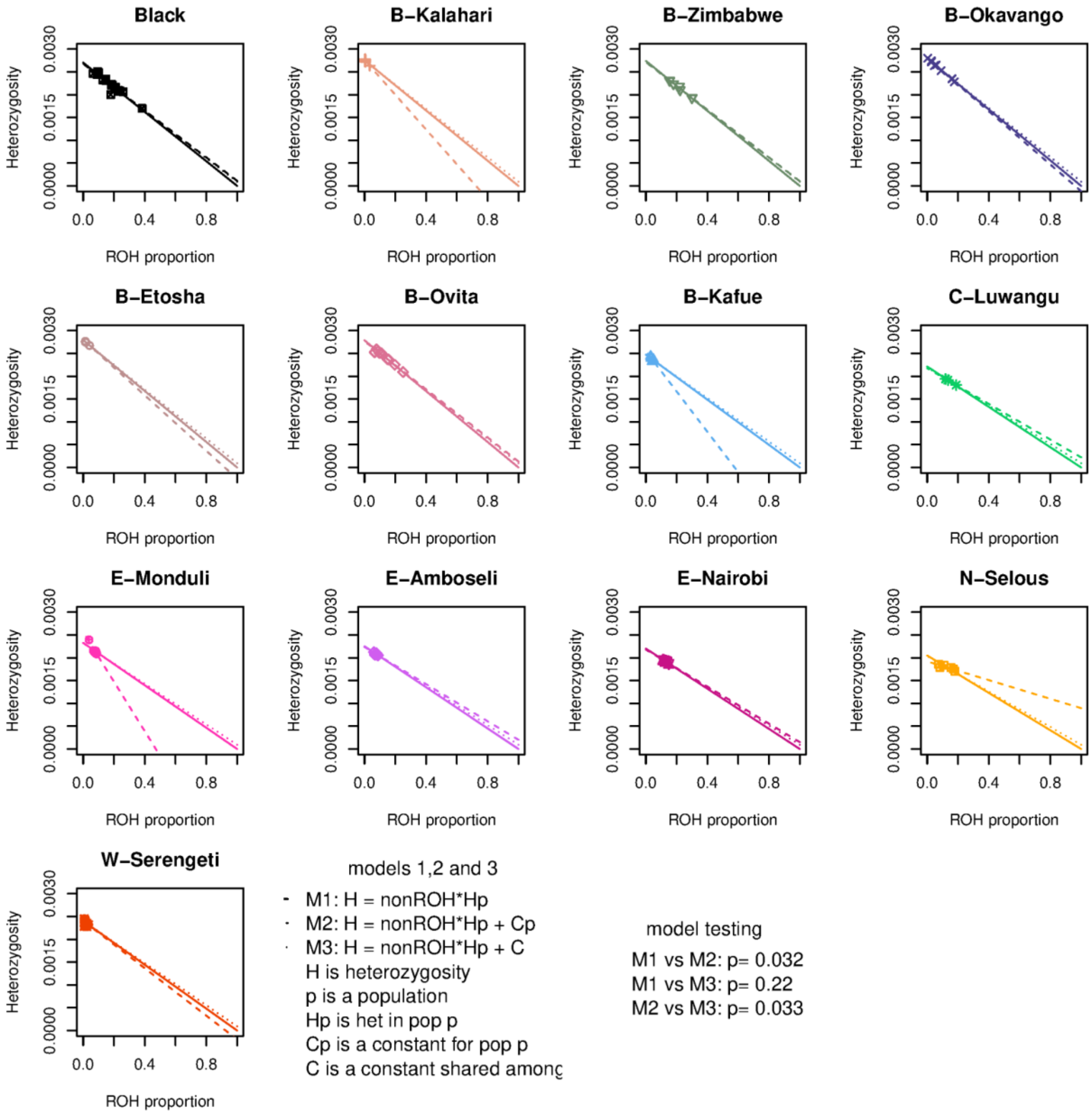

Supplementary Figure 12. Heterozygosity and ROH proportion for wildebeest populations. Each panel shows the relationship between ROH and heterozygosity within each population. Linear regression was used to fit lines for the population with the constraint of the lines intercepting the x-axis at 1 (0% heterozygosity for 100% ROH).

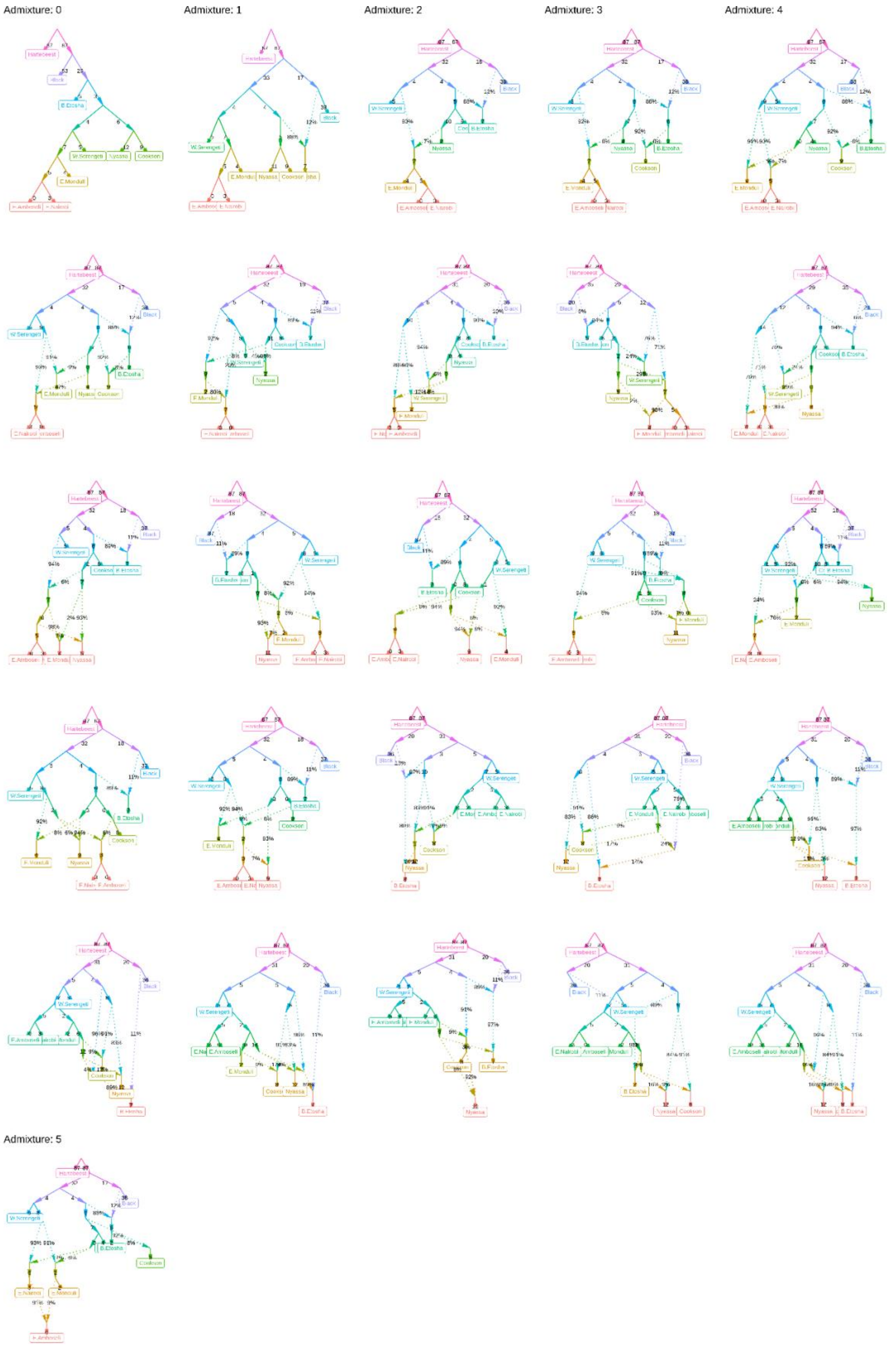
Admixture: 0
Admixture: 1
Admixture: 2
Admixture: 3
Admixture: 4
Admixture: 5

Supplementary Figure 13. Statistically non-rejected admixture graphs with the number of admixture events ranging from 0 to 5 in qpGraph. For a given number of admixture events, admixture graphs whose likelihood scores are not significantly worse than the best graph are included and plotted. In each graph, branch lengths (genetic drift) are shown besides arrows of solid lines and admixture proportions are shown besides arrows of dashed lines. See the summary of these admixture graphs in Supplementary Data 4.

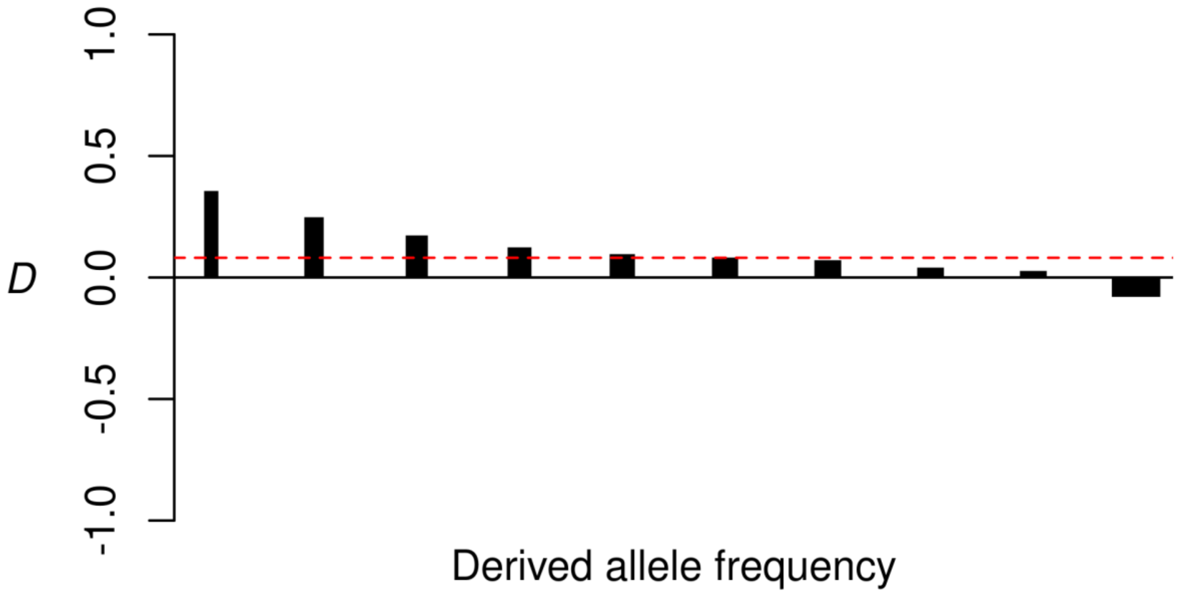

Supplementary Figure 14. Frequency spectrum of D-statistics (D_{FS}) between the blue wildebeest populations and the black wildebeest. To calculate *D*-statistics, we used the western white-bearded (W-Serengeti) population as H1, brindled population in Etosha (B-Etosha) as H2, black wildebeest as H3 and hartebeest as H4 (outgroup). Vertical lines indicate the stratified *D* values for different bins of derived allele frequency; the number of bins is equal to the number of haploid samples in H1 and H2 (5 diploid samples). We found positive *D* values at low derived frequency. Widths of vertical lines are drawn in proportion to their weighting. The horizontal dashed line indicates the global *D* value.

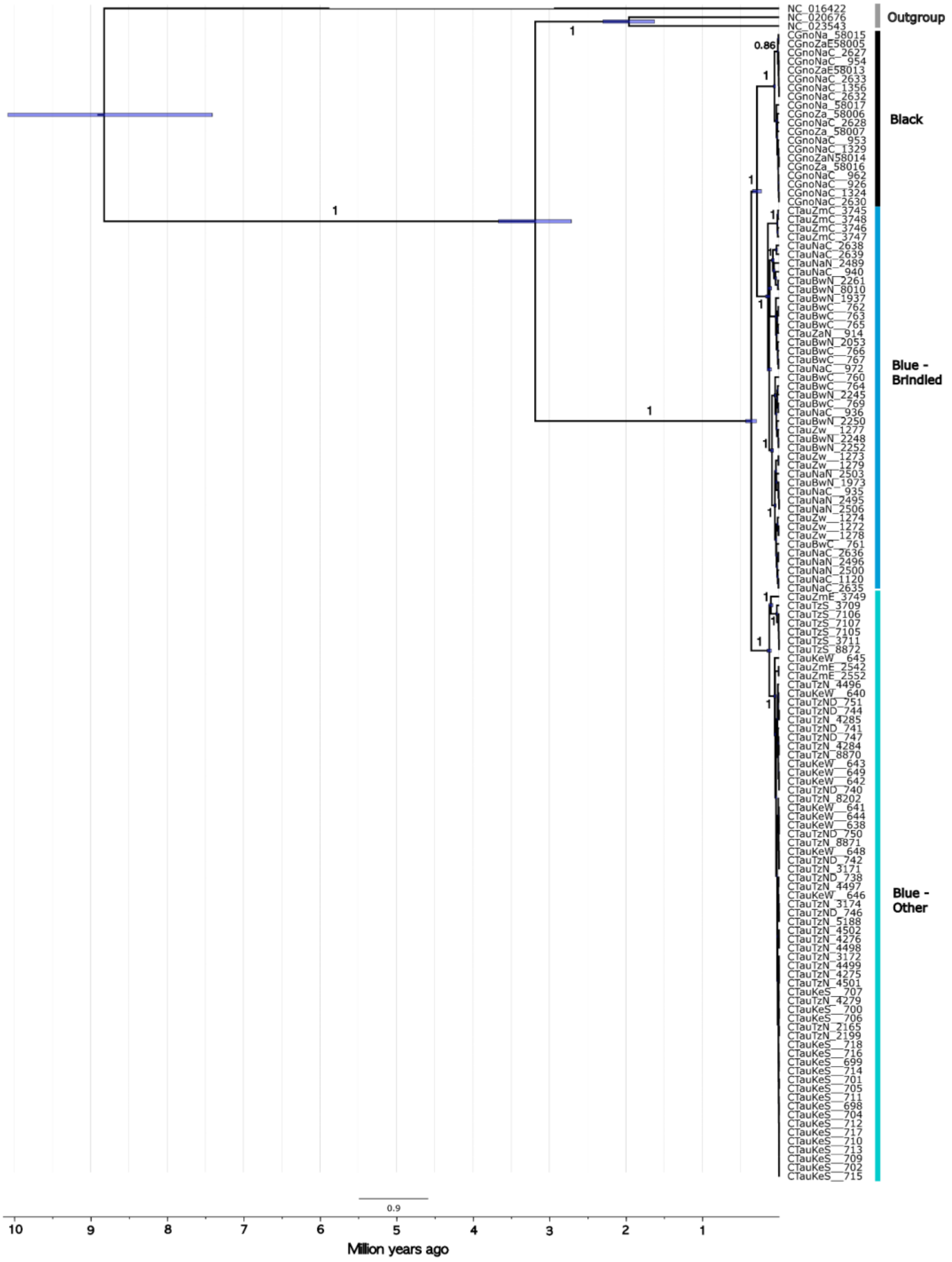

Supplementary Figure 15. Phylogenetic tree using mitochondrial DNA including wildebeest and outgroups. Phylogenetic tree was constructed using BEAST2 (for more details see 'mtDNA analyses and phylogenetic tree' in Methods).

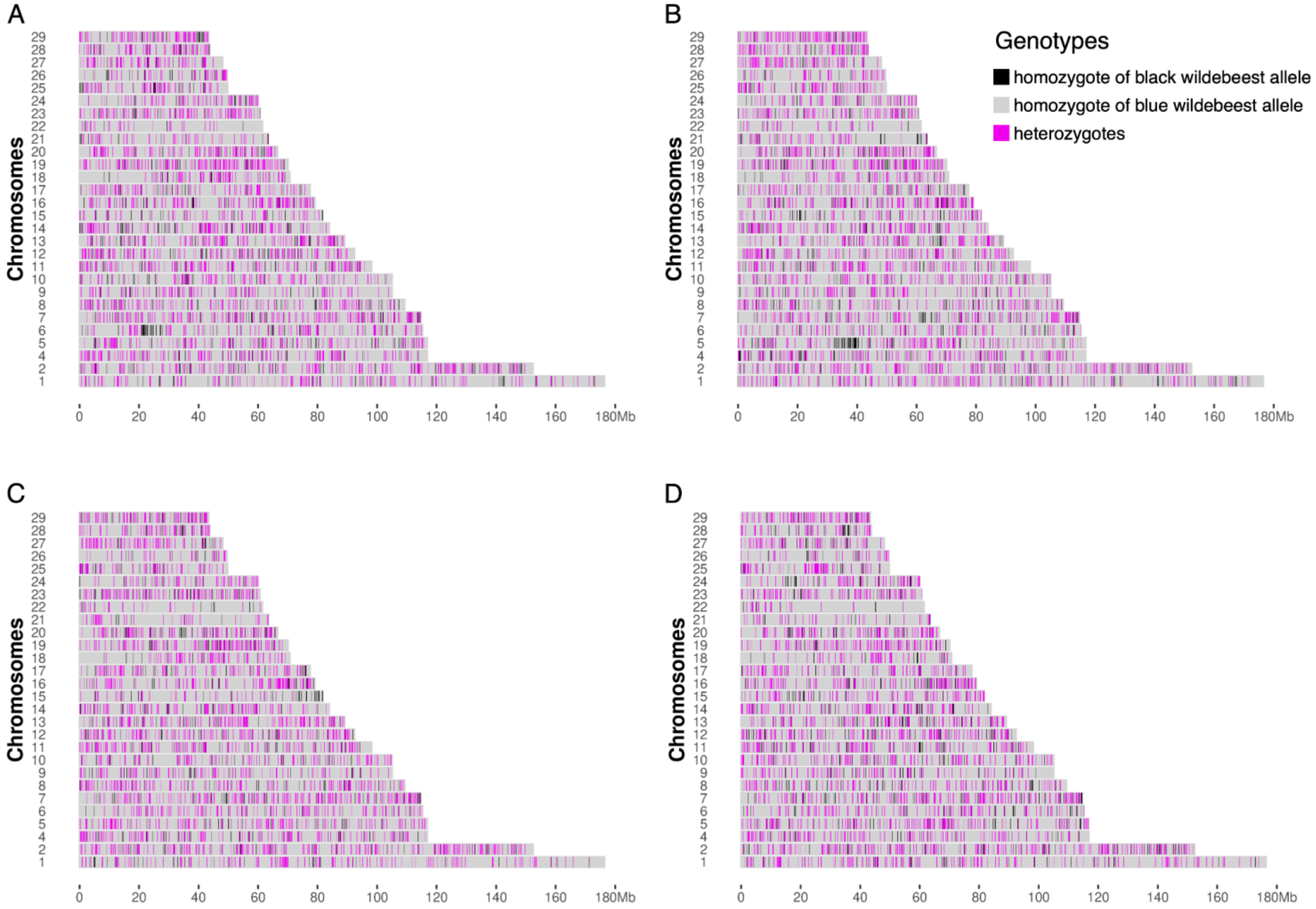

Supplementary Figure 16. Local ancestries inferred using LOTER in all samples of the brindled population in Etosha (B-Etosha) excluding the one shown in Fig. 3B. The black wildebeest population and Western white-bearded population were used to represent the ancestry of black wildebeest (black bar) and blue wildebeest (gray bar), respectively. The heterozygous ancestries are indicated by purple bars.

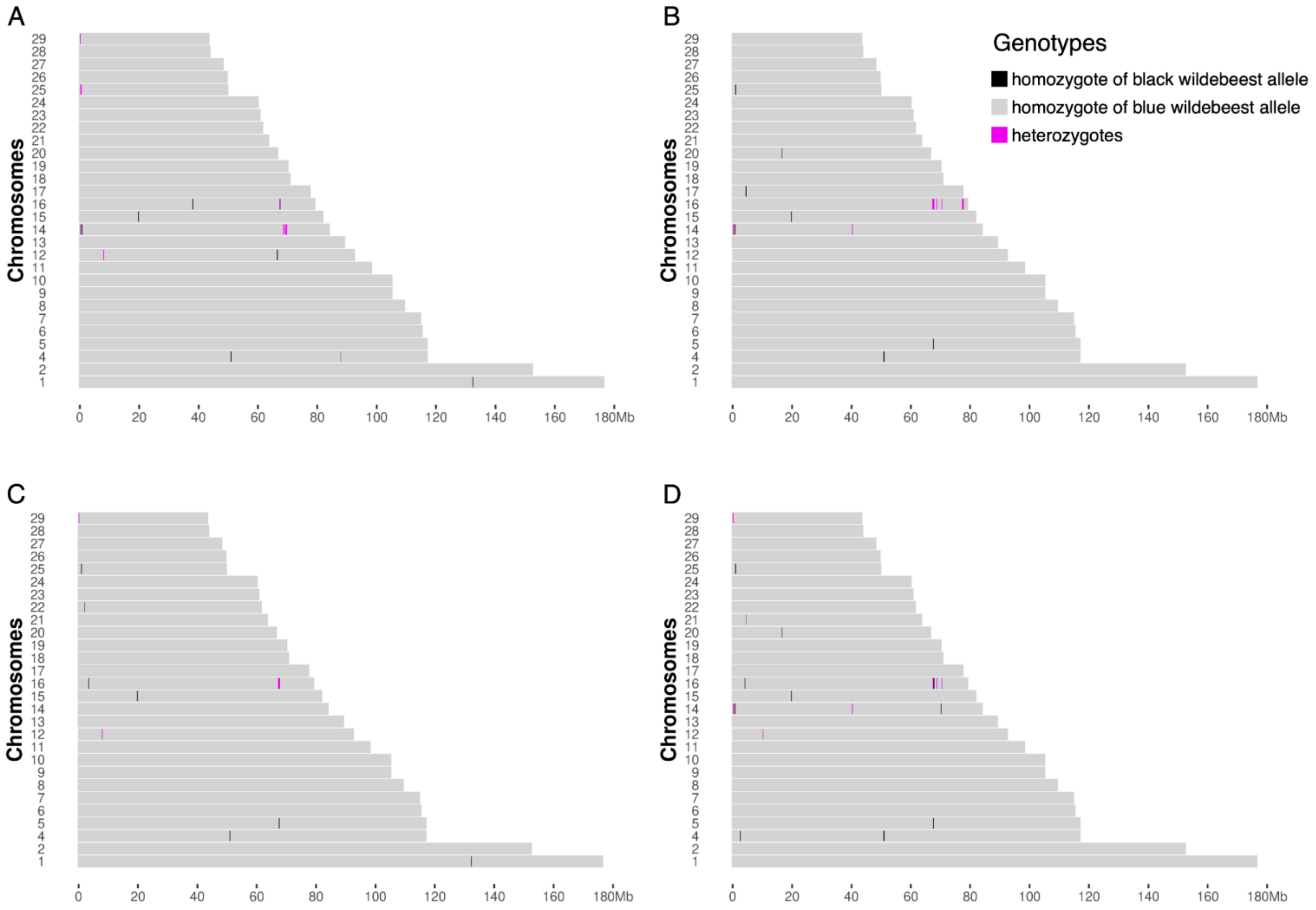

Supplementary Figure 17. Local ancestries inferred using LOTER in all samples of the Nyassa population excluding the one shown in Fig. 3B. The black wildebeest population and Western white-bearded population were used to represent the ancestry of black wildebeest (black bar) and blue wildebeest (gray bar), respectively. The heterozygous ancestries are indicated by purple bar

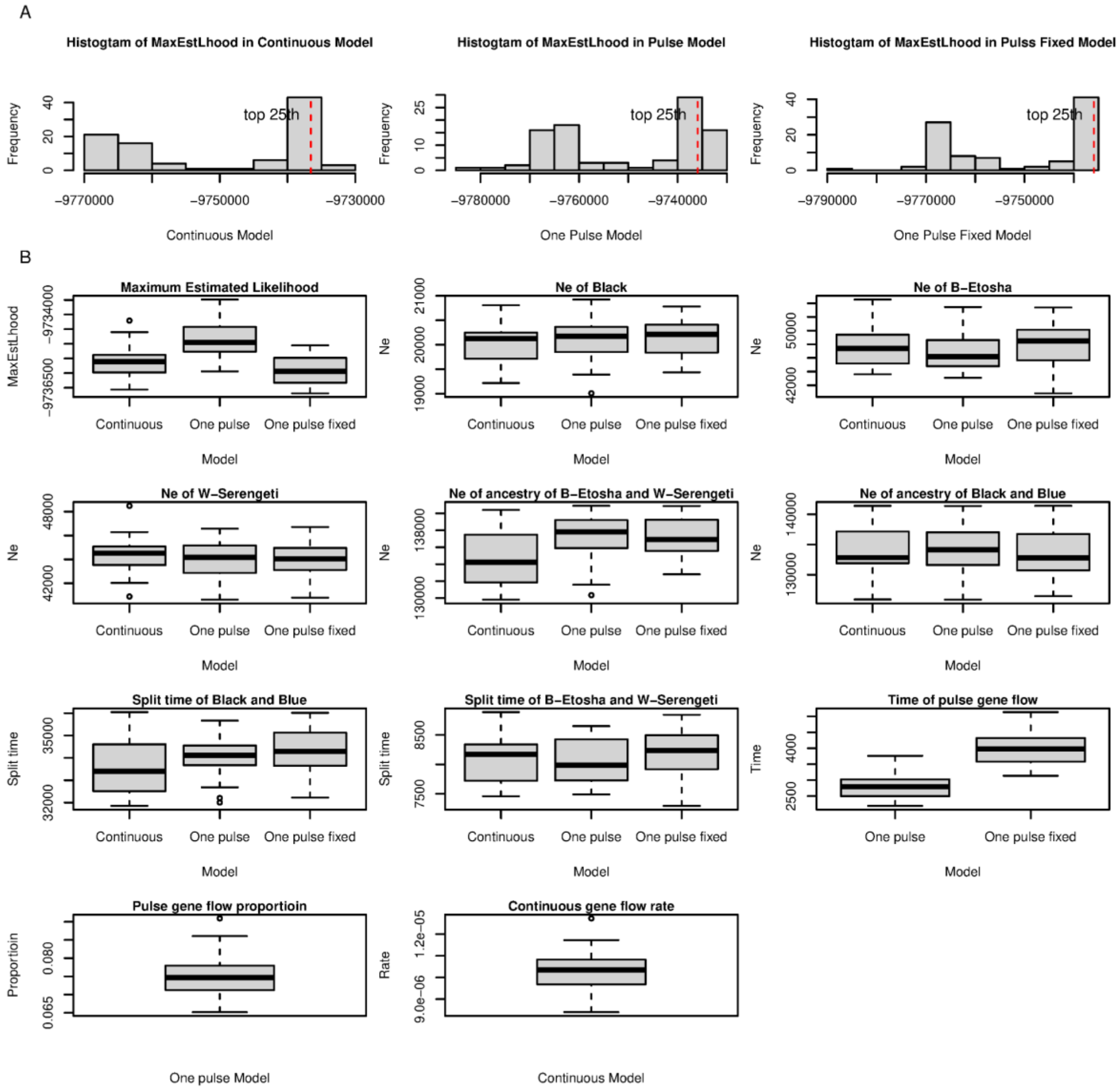

Supplementary Figure 18. Likelihoods and parameter point estimates of the fastsimcoal2 demographic models.

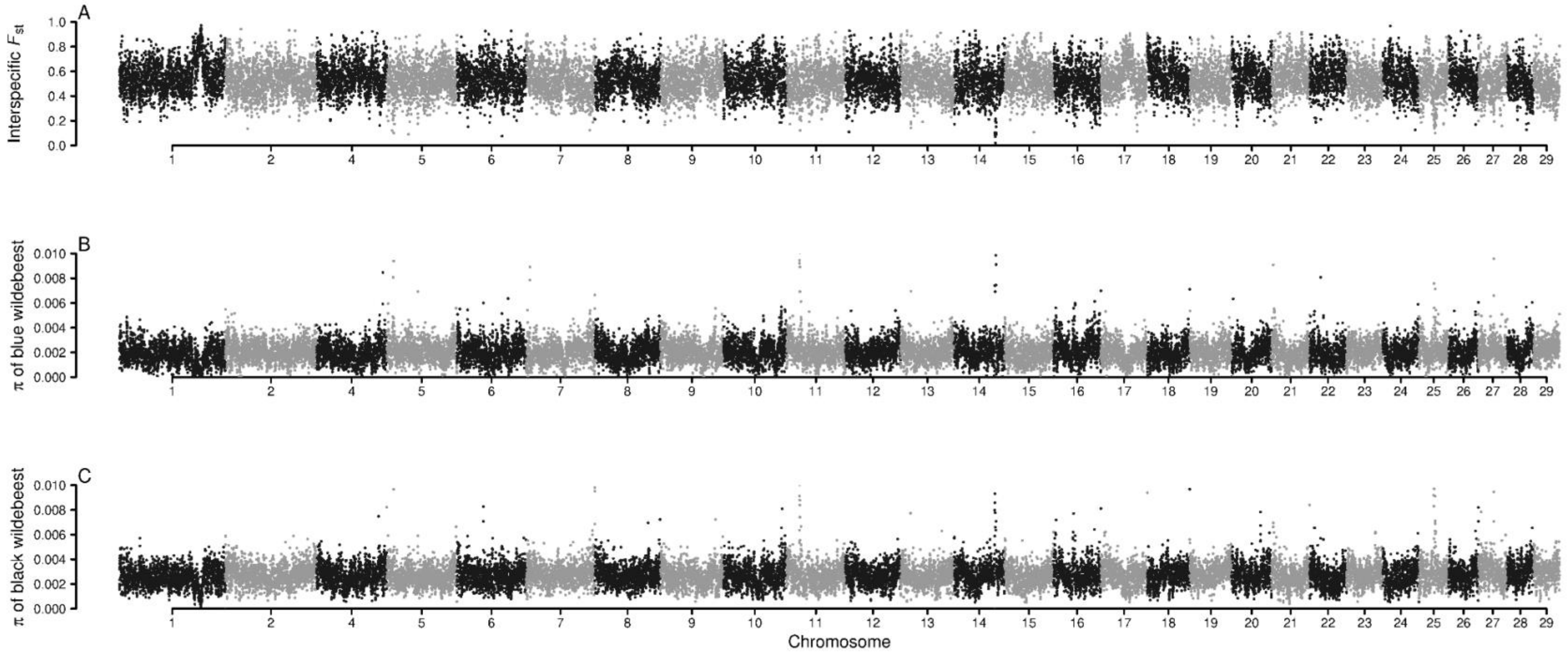

Supplementary Figure 19. Genomic landscapes of differentiation and nucleotide diversity in wildebeest. (**A**) Patterns of genetic differentiation between the black wildebeest and the Brindled population in Etosha– a representative population of the blue wildebeest are measured by Hudson's F_{ST} based on non-overlapping windows of 100k bases. Patterns of genetic diversity in the blue wildebeest (Brindled population in Etosha, (**B**) and black wildebeest (**C**) are measured using nucleotide diversity ($\boldsymbol{\pi}$). Notably, there is a highly differentiated region (F_{ST}) on chromosome 1, where both species exhibit remarkably reduced nucleotide diversity (see Fig. S20 below).

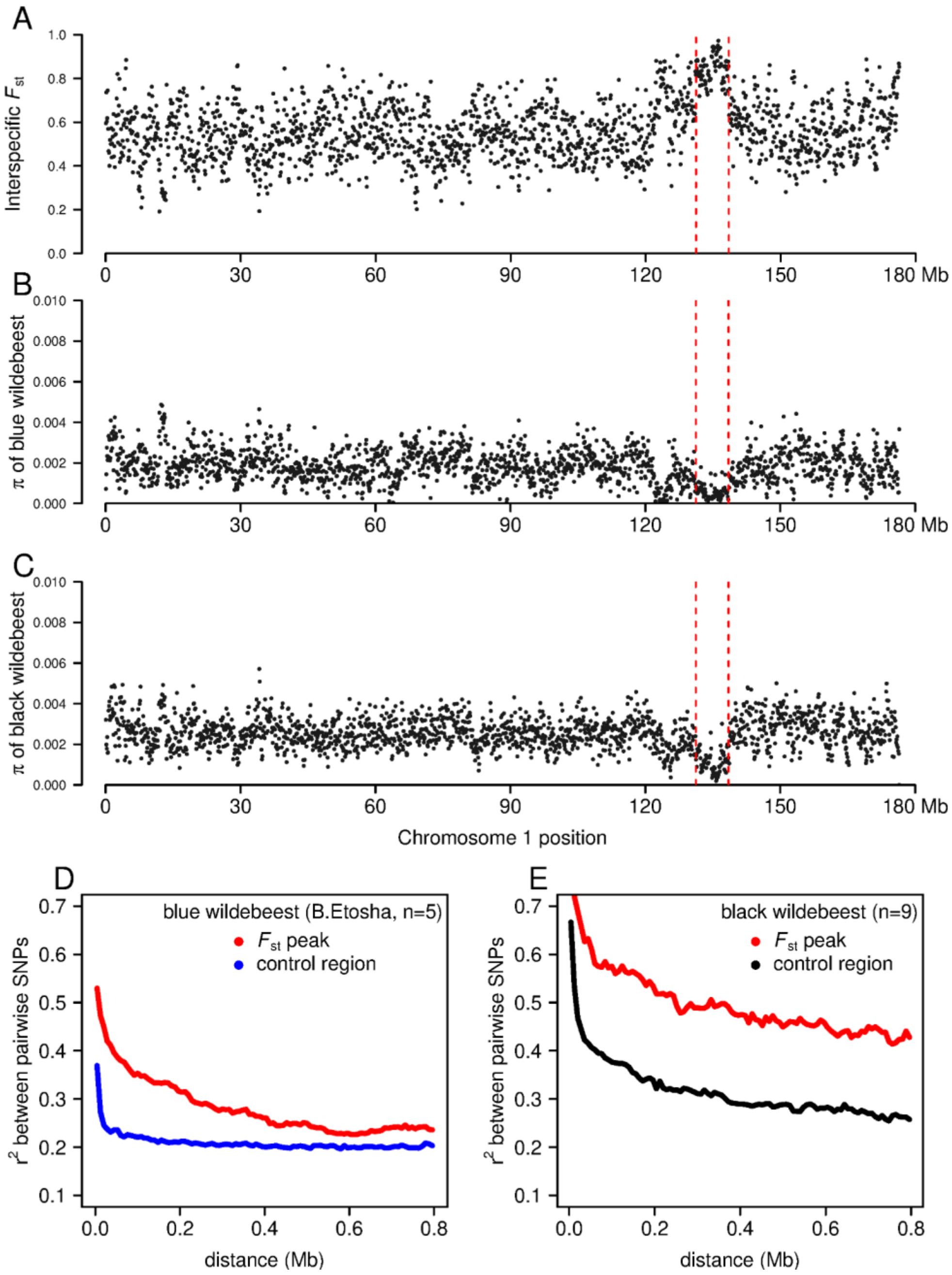

Supplementary Figure 20. Characterization of the highly differentiated region on chromosome 1. (**A**) The genomic block spanning from 131,200,000 to 138,500,000 bp (highlighted by the dashed line in red) on chromosome 1 is highly differentiated between the blue (represented by the Brindled population in Etosha) and black wildebeest based on F_{ST} scan. In this region, both species exhibit decreased nucleotide diversity (**B** and **C**). In addition, the block of F_{ST} peak is also characterized by elevated linkage disequilibrium compared to the remaining regions on chromosome 1 in both species (**D** and **E**).

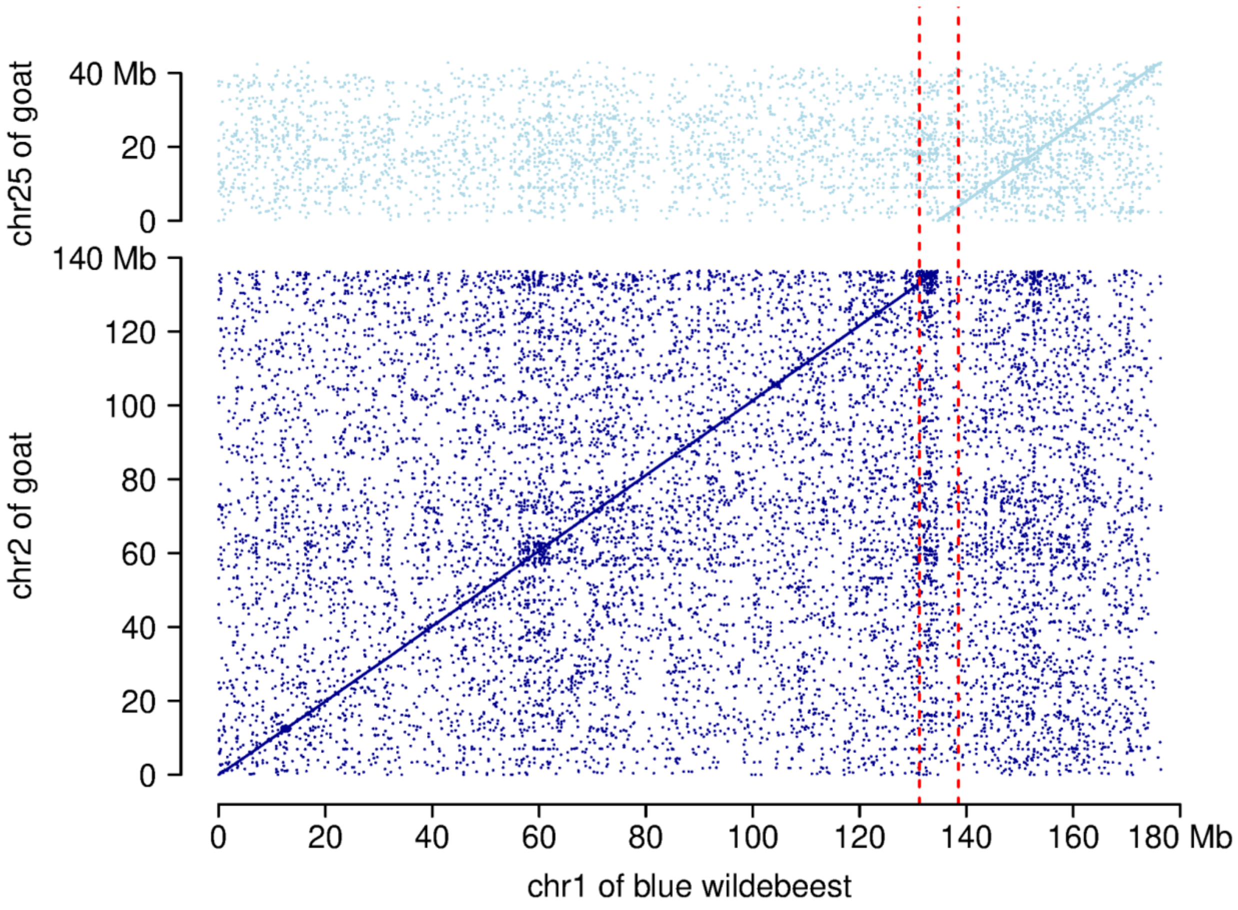

Supplementary Figure 21. Alignment between chromosome 1 of wildebeest and chromosomes 2, 25 of domestic goat. Pairwise sequence alignment between the blue wildebeest and domestic goat genomes using lastz shows that chromosome 1 of blue wildebeest corresponds to chromosome 2 (dark blue) and chromosome 25 (light blue) of goat, in line with [1], which reports that chromosome 1 of blue wildebeest is a product of fusion of two ancestral chromosomes in bovine. The interval between the dashed red lines indicates the region of F_{ST} peak between the blue and black wildebeest, spanning from 131,200,000 to 138,500,000 bp on chromosome 1 of blue wildebeest geno

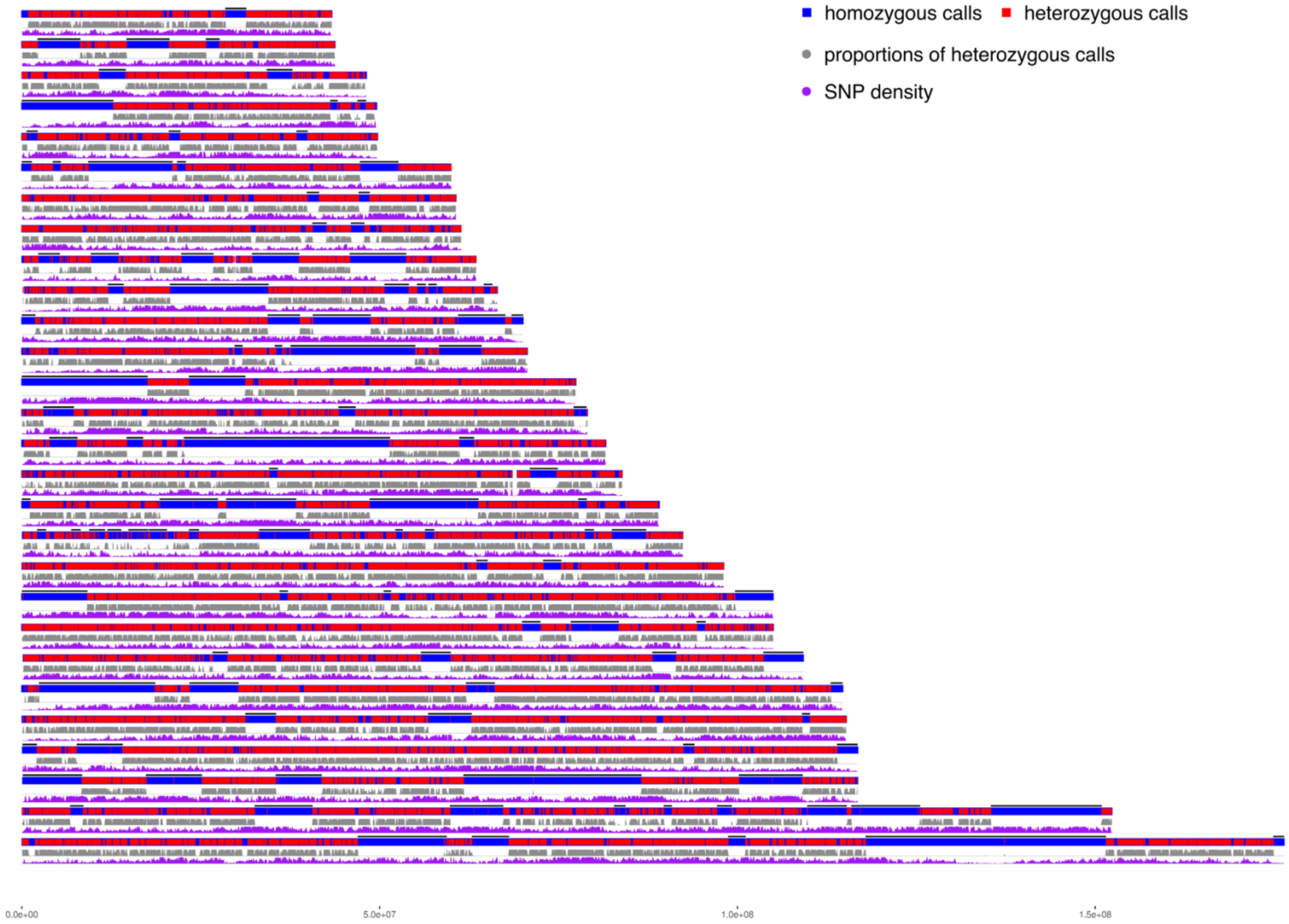

Supplementary Figure 22. Genotype calls, proportions of heterozygous sites and SNP density for ROH validation in a black wildebeest sample (CGnoNaC_2630). For each chromosome, blue vertical bars indicate homozygous sites, and red vertical bars are superimposed to show heterozygous sites. Identified ROHs are marked by black horizontal bars. The gray line below homozygous/heterozygous calls shows proportions of heterozygous calls in a window of 100 kb. The purple line below shows the number of SNPs along the 100 kb window.

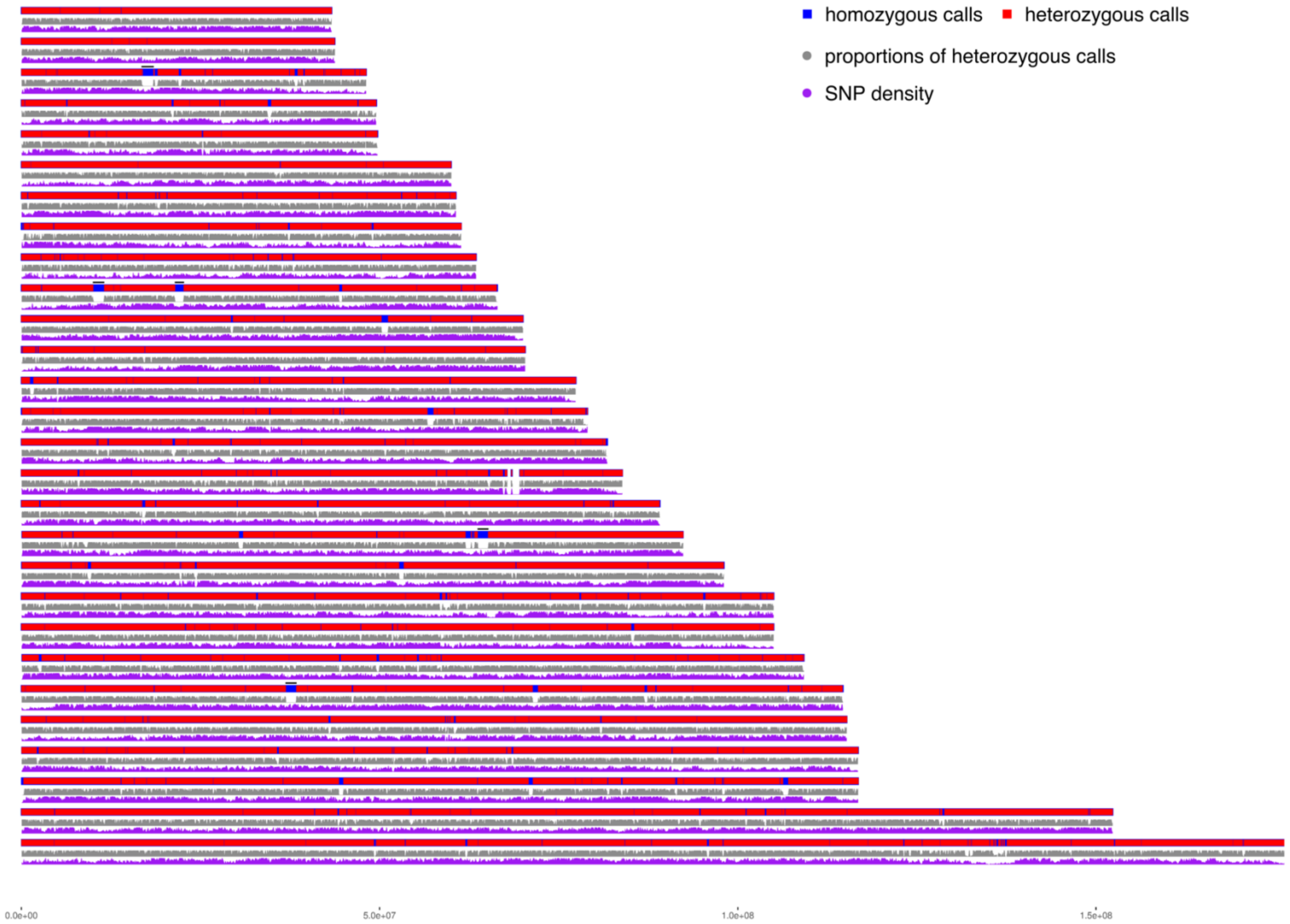

Supplementary Figure 23. Genotype calls, proportions of heterozygous sites and SNP density for ROH validation in a Brindled wildebeest sample (CTauBwC__761). For each chromosome, blue vertical bars indicate homozygous sites, and red vertical bars are superimposed to show heterozygous sites. Identified ROHs are marked by black horizontal bars. The gray line below homozygous/heterozygous calls shows proportions of heterozygous calls in a window of 100 kb. The purple line below shows the number of SNPs along the 100 kb window.

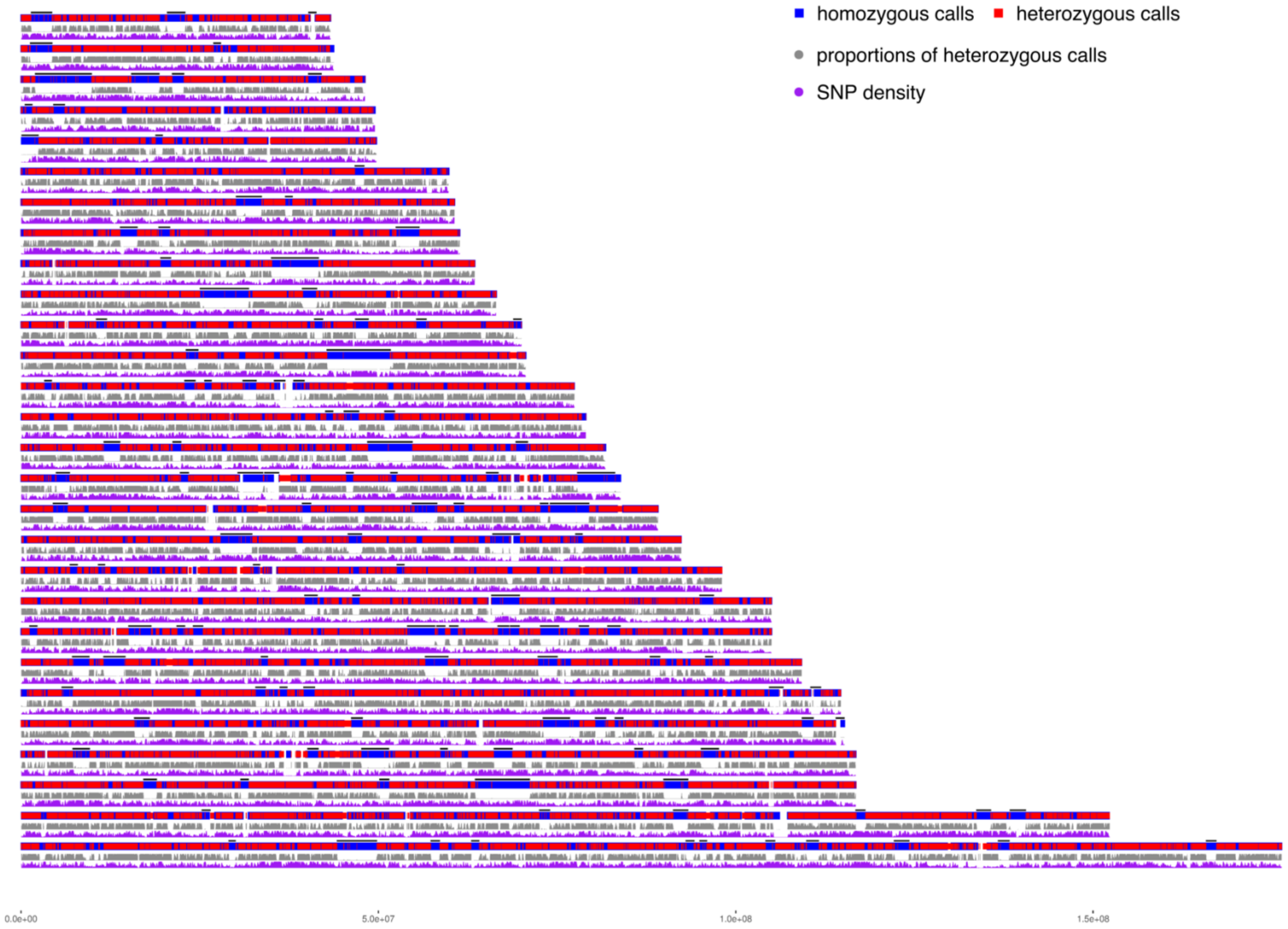

Supplementary Figure 24. Genotype calls, proportions of heterozygous sites and SNP density for ROH validation in a Cookson wildebeest sample (CTauZmE_2542). For each chromosome, blue vertical bars indicate homozygous sites, and red vertical bars are superimposed to show heterozygous sites. Identified ROHs are marked by black horizontal bars. The gray line below homozygous/heterozygous calls shows proportions of heterozygous calls in a window of 100 kb. The purple line below shows the number of SNPs along the 100 kb window.

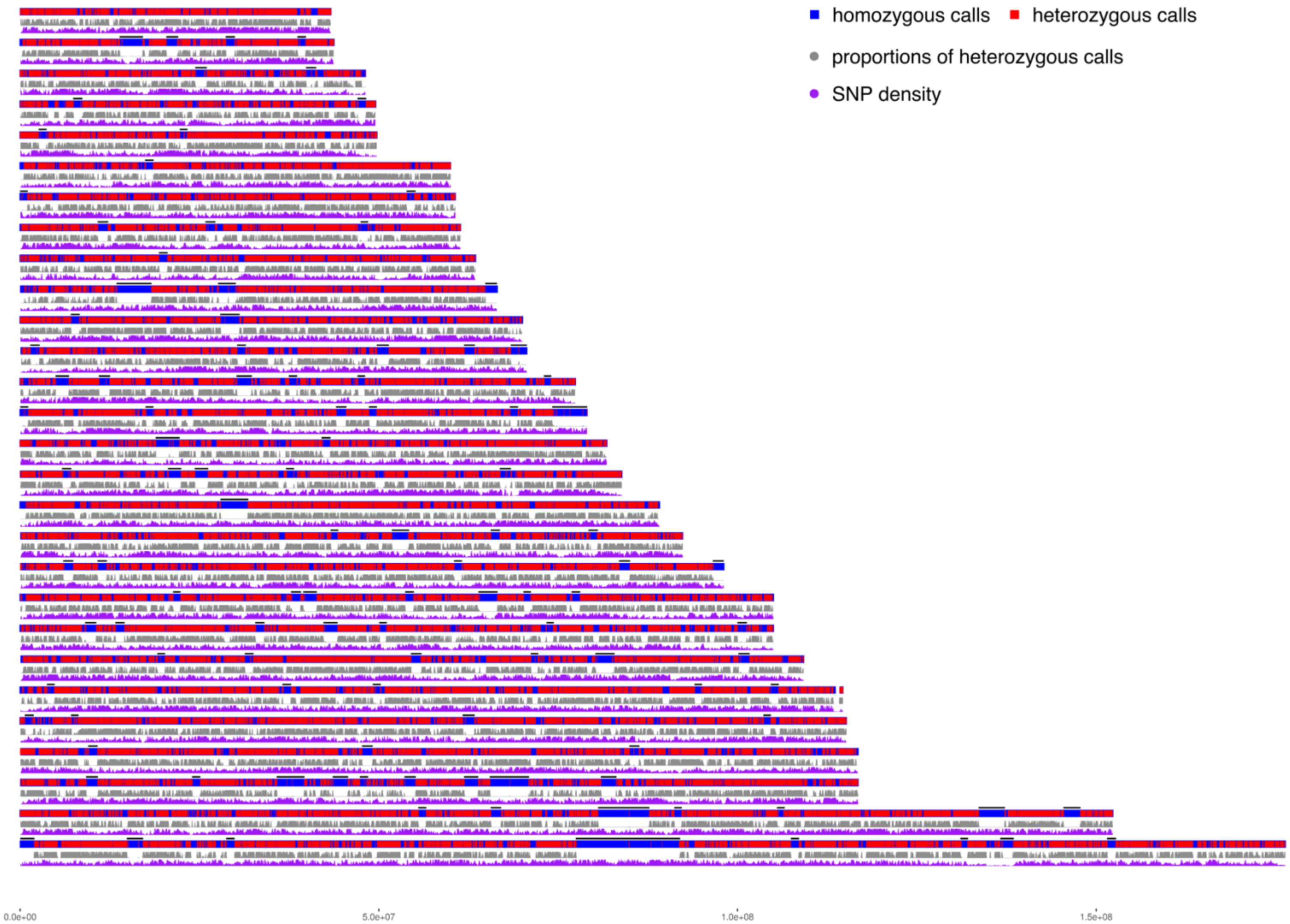

Supplementary Figure 25. Genotype calls, proportions of heterozygous sites and SNP density for ROH validation in a Nyassa wildebeest sample (CTauTzS_3711). For each chromosome, blue vertical bars indicate homozygous sites, and red vertical bars are superimposed to show heterozygous sites. Identified ROHs are marked by black horizontal bars. The gray line below homozygous/heterozygous calls shows proportions of heterozygous calls in a window of 100 kb. The purple line below shows the number of SNPs along the 100 kb window.

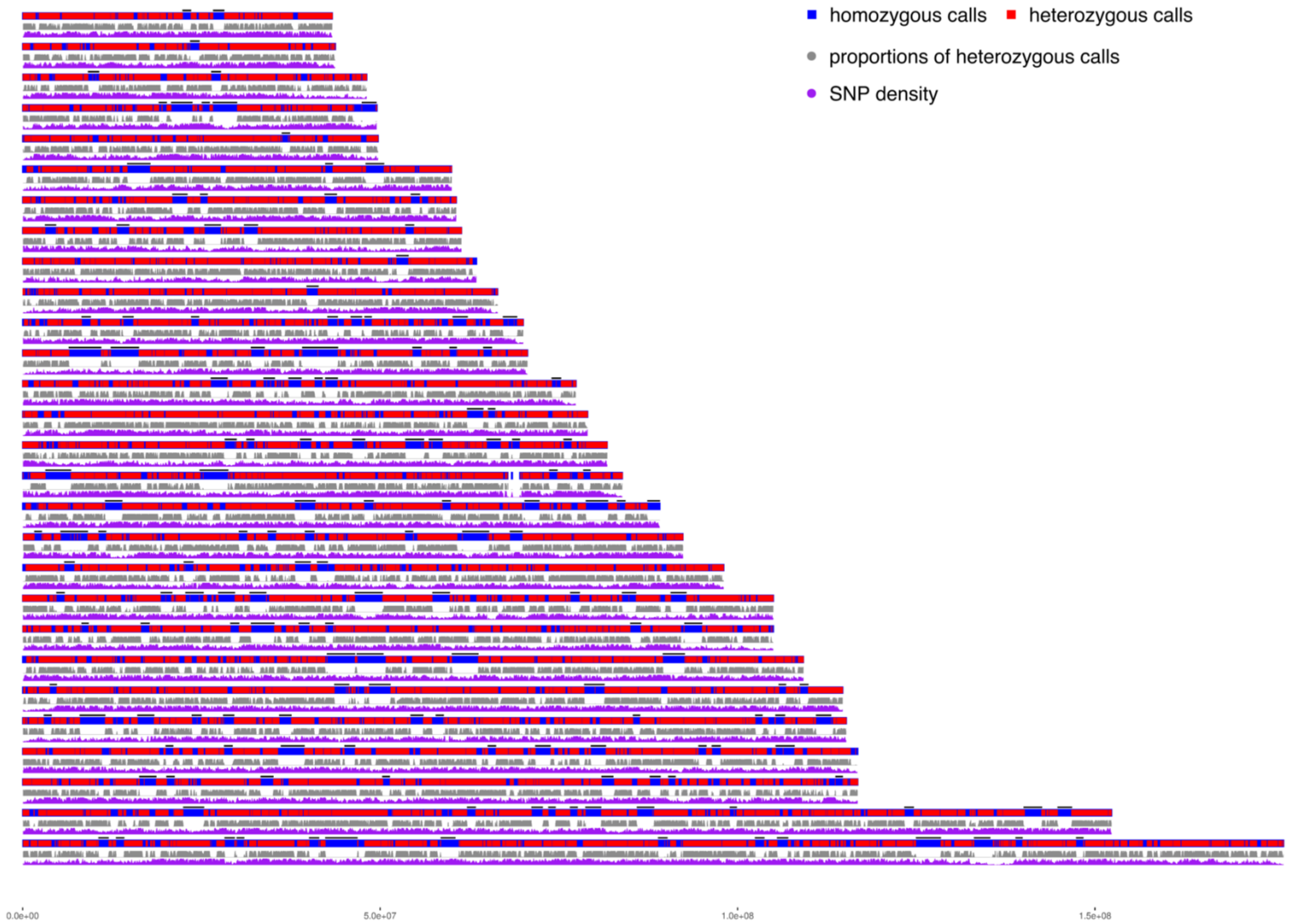

Supplementary Figure 26. Genotype calls, proportions of heterozygous sites and SNP density for ROH validation in an Eastern white-bearded wildebeest sample (CTauKeS__716). For each chromosome, blue vertical bars indicate homozygous sites, and red vertical bars are superimposed to show heterozygous sites. Identified ROHs are marked by black horizontal bars. The gray line below homozygous/heterozygous calls shows proportions of heterozygous calls in a window of 100 kb. The purple line below shows the number of SNPs along the 100 kb window.

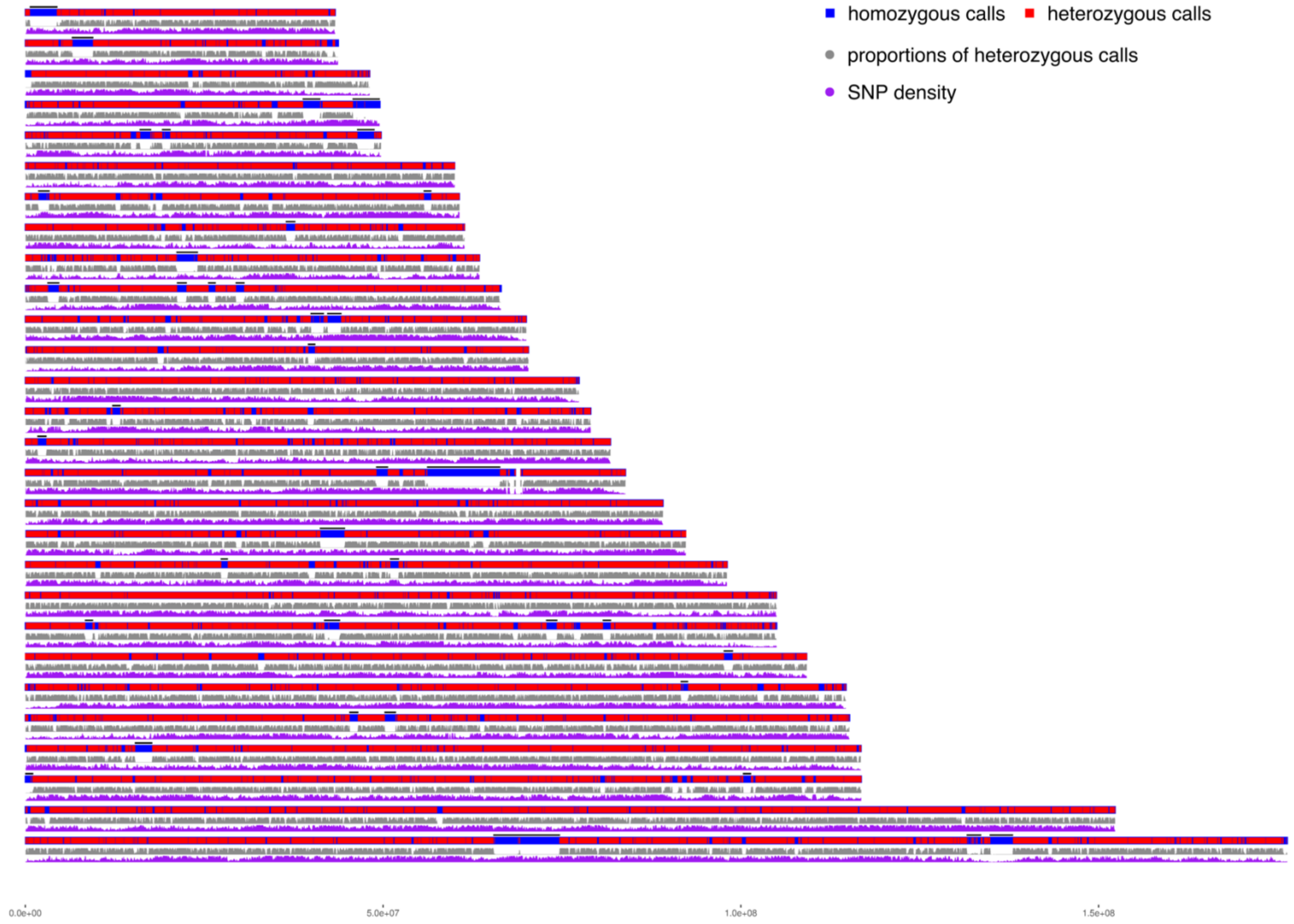

Supplementary Figure 27. Genotype calls, proportions of heterozygous sites and SNP density for ROH validation in a Western white-bearded wildebeest sample (CTauKeW__649). For each chromosome, blue vertical bars indicate homozygous sites, and red vertical bars are superimposed to show heterozygous sites. Identified ROHs are marked by black horizontal bars. The gray line below homozygous/heterozygous calls shows proportions of heterozygous calls in a window of 100 kb. The purple line below shows the number of SNPs along the 100 kb wind

Supplementary Note

Supplementary Note 1. Population homogeneity criteria. We used an ad-hoc approach to assess the homogeneity of the 12 clusters of blue wildebeest population inferred from the ADMIXTURE analyses. evalAdmix showed very limited correlations of residuals within the six populations representing the four subspecies- Cookson's, Nyassa, Eastern and Western white-beared wildebeest at K=12 (Fig. 1C), indicating high genetic homogeneity of these population units. We further exclude one sample exhibiting noticeable signs of recent admixture in both the Eastern white-bearded population in Tanzania (E-Monduli) and Nyassa (Fig. 1C). Among the brindled wildebeest, which showed complicated population structures, only two populations had almost no correlations of residuals in evalAdmix, including the Zambia (B-Kafue) and the long-range panmictic group around the Kalahari desert (B-Kalahari). In contrast, significant pairwise correlations of residuals were observed in the populations from Botswana north (B-Okavango), Namibia north (B-Etosha), central Namibia (B-Ovita) and Zimbabwe (B-Zimbabwe). This suggests either relatedness or substructure within the clusters. Among these four populations with poor model fit, positive correlations of residuals in B-Etosha were caused by a pair of half siblings. After removing one sample of the related pair, a new evalAdmix analysis including only the Brindled wildebeest showed no or very low correlations of residuals in B-Etosha, but still notable correlations of residuals in B-Okavango, B-Ovita and B-Zimbabwe. In addition, we also removed one sample showing admixed ancestry in B-Kalahari. Taken together, we identified a total of nine populations with high genetic homogeneity in the blue wildebeest, including B-Etosa (n=5), B-Kafue (n=4), B-Kalahari (n=11), C-Selous (n=3), N-Luangwa (n=5), E-Amboseli (n=9), E.Monduli (n=10), E. Nairobi (n=10) and W-Serengeti (n=28).

The black wildebeest samples exhibited no clear discrete structure based on ADMIXTURE (K=2–4, Fig. S7). Furthermore, evalAdmix showed no negative or positive mean correlation within or between sampling locations, suggesting that a higher K is not supported (Fig. S8). However, many individuals show positive and negative correlations of residuals, indicating the presence of sub-structure within the black wildebeest. Therefore, based on genetic ancestry and geographical coherence, we defined a homogenous black population consisting of a subset of the samples from Namibia. We further evaluated homogeneity of the defined population using the ABBA-BABA test (also known as D-statistics), in which H1 and H2 were pairwise individuals of the black population, H3 was the Brindled population around the Kalahari desert (B-Kalahari) and H4 had two hartebeest samples. We identified one outlier in the ABBA-BABA test, which was excluded from the final set of the clean black population (n=9).

To further assess homogeneity of the nine homogenous blue wildebeest populations and one black wildebeest population, we inspected the patterns of decay of linkage disequilibrium (LD). As

expected for a population composed of individuals of high genetic uniformity, LD curves plateau and stabilize to a constant value with a few Mb for all the nine blue wildebeest populations. However, the black wildebeest population plateaued at a higher value, which was likely driven by the severe bottleneck experienced in the 1900s. Non-homogenous populations of blue wildebeest- B-Ovita and B-Zimbabwe, exhibited similar patterns as the black wildebeest, suggesting possible substructure within them (Fig. S6).

The final selection of homogenous populations includes only individuals sequenced on the MGI platform. Although we did not see any indication of batch bias, this selection of individuals also rules out the possibility of batch bias caused by the sequencing platform.

Supplementary Note 2. Historical records and identification of migratory and non-migratory populations in eastern and southern Africa. Blue wildebeest populations exhibit a continuum of migratory habits ranging from completely sedentary through vagrant (periodically, sometimes seasonally, moving relatively short distances mainly in response to drinking or feeding availability) to full-blown nomadic (obligatorily performing long-distance yearly migrations over 100s of kilometers). In the following, we highlight two regions in which wildebeest are or are known to have formerly been highly migratory, i.e. migrate or used to migrate over 100s of kilometers every year.

Western and Eastern white bearded wildebeest

To the east of the Great (Gregory) Rift Valley, there is reliable historical evidence that the eastern white-bearded wildebeests formerly maintained spatially large and connected migratory population networks [2,3], whereas these populations today are nearly completely isolated from each other[4]. Documented disruption of migration routes in the distribution range of the eastern white-bearded wildebeest include a break of connectivity in Kajiado County in Kenya, formerly connecting the Nairobi Park and the Athi-Kaputiei Plains population to the Amboseli ecosystem, Ngong National Reserve; Nairobi, Thika and Ruiru; Ithanga Hills, Voi and Tana River; Lake Natron and across the Rift Wall to Narok County and thence to the eastern shores of Lake Naivasha, and reaching south into Tanzania during particularly dry periods[3,5–9]. As a result, wildebeest migration has completely collapsed in the Athi-Kaputiei and the Amboseli Ecosystems in Kajiado County of Kenya [5–7]. Other migration routes east and west of Mt. Meru formed a historical connection involving the Tarangire Ecosystem in northern Tanzania[2]. In contrast, the western white-bearded population, inhabiting the Masai Mara on the Kenyan side and the Serengeti on the Tanzanian side, remains an example of a nearly intact and unimpeded large migratory wildebeest population[10], even though migrations of some of its sub-populations, such as the Mara-Loita and the Mara-Loita-Ilkerin-Loliondo migrations, have recently collapsed[11] .

Brindled wildebeest

In southern Africa, archived records show that the brindled wildebeest in and around the Kalahari desert had until as late as 1980 been highly migratory[12], possibly also interconnecting with migratory populations in the Okavango area and parts of northern Namibia [4]. This accords with our result that the brindled samples from a number of localities spanning a large area roughly consistent with the outline of the Kalahari basin form a single genetic population. The Kalahari population shows the same genetic migration syndrome as the Serengeti population when compared with the other non-migratory brindled wildebeest populations. In this population, wildlife fences erected between 1958 and 2000 to prevent wildlife from moving into livestock ranges are known to have led to serious ecological consequences for migratory species including

wildebeest [12–14]. Our samples from this area were collected between 1992-1995, and in our data this population still retains the genetic features characteristic of a migratory wildebeest population, such as higher heterozygosity, fewer ROHs and long-distance genetic connectivity. Similarly, also the brindled wildebeest population in Etosha was a migratory population until recently, but encompassing a smaller geographical range than the two examples above[4]. This population also clearly shows the genetic migration syndrome.

It was not within the scope of our study to perform a detailed classification of blue wildebeest populations along this continuum. Instead, we have defined populations that inhabit the two areas of the blue wildebeest range where all sources agree on the existence of long-distance wildebeest migrations until circa 1960 as ‘migratory’, and all other populations as ‘non-migratory’. Our genetic results showing the presence of a ‘migration syndrome’ suggest that such a distinction, while a simplification of a more complex range of migratory conditions, indeed has some merit.

In the following we quote excerpts from Estes & East (2009)[3] that support our definitions of current and former migratory populations of blue wildebeest (our emphasis in bold):

Eastern white bearded (p. 72)
Like the gnu populations of the northern **Kalahari** region, to which the Masai steppe is comparable, the main population of eastern Masailand was **nomadic and migratory**. There were also several small satellite populations with sedentary habits in areas with permanent water. In pre-colonial days, the open grassland and Acacia savanna which covered virtually the whole of Masailand formed the wet-season dispersal area for wildebeest, zebra, and Thomson’s gazelle, the three most numerous and most migratory plains species.[...] The amount of game that inhabited eastern Masailand in former times is unknown. Considering that the Serengeti region currently carries over a million head of large mammals, the **far larger area of eastern Masailand could presumably have carried at least as many**. Surely the gnu population must have numbered at least 100,000 at the end of the 19th century.

Eastern white bearded (p. 95)
The eastern white-bearded wildebeest **formerly ranged widely** over the open grasslands and acacia savannas of the Masai steppe in northern Tanzania, to the east of the Gregory Rift Valley. At the end of the 19th century, virtually **the whole of eastern Masailand was probably a wet-season dispersal area for migratory plains game**, which concentrated around permanent sources of water during the dry season; though numbers are unknown, there were probably at least hundreds of thousands of wildebeest.

Kalahari population (pp89-90)
The Kalahari Desert, which occupies most of Botswana, the northern Cape of South Africa, and eastern Namibia, formerly supported one of Africa’s great plains-game ecosystems. [...] During

the wet season, a **large migratory wildebeest population** dispersed throughout the Kalahari savannas. When these areas became waterless for several months during the dry season, and especially in severe drought years, the wildebeest concentrated near permanent water in areas such as the Makgadikgadi Pans, the Lake Ngami depression and the Chobe River in Botswana, the floodplains bordering the **Okavango** Swamp in Botswana and Namibia, **Etosha** Pan, Ovamboland and the Caprivi Strip in Namibia, along the Cunene and Cubango Rivers in Angola, and formerly along the Limpopo River in Botswana and South Africa, and the Orange River in **South Africa**. This vast region may have been occupied by **a single wildebeest population**, which dispersed widely during the wet season but broke up into separate concentrations during the dry season. Its size will never be known, but it must have comprised **at least several hundred thousand individuals**.

Supplementary References

1. Vozdova, M. *et al.* A comparative study of meiotic recombination in cattle (*Bos taurus*) and three wildebeest species (*Connochaetes gnou*, *C. taurinus taurinus* and *C. t. albojubatus*). *CGR* **140**, 36–45 (2013).
2. Lamprey, H. F. Estimation of the large mammal densities, biomass and energy exchange in the tarangire game reserve and the Masai steppe in Tanganyika. *Afr. J. Ecol.* **2**, 1–46 (1964).
3. Msoffe, F. U. *et al.* Wildebeest migration in East Africa: Status, threats and conservation measures. *bioRxiv* 546747 (2019) doi:10.1101/546747.
4. Estes, R. & East, R. *Status of the wildebeest (Connochaetes taurinus) in the wild 1967-2005*. (Wildlife Conservation Society, 2009).
5. Ogutu, J. O. Changing wildlife populations in Nairobi National Park and adjoining athi-kaputiei plains: Collapse of the migratory wildebeest. *Open Conserv. Biol. J.* **7**, 11–26 (2013).
6. Ogutu, J. O., Piepho, H.-P., Said, M. Y. & Kifugo, S. C. Herbivore dynamics and range contraction in Kajiado County Kenya: climate and land use changes, population pressures, governance, policy and human-wildlife conflicts. *The Open Ecology Journal* **7**, (2014).
7. Said, M. Y. *et al.* Effects of extreme land fragmentation on wildlife and livestock population abundance and distribution. *J. Nat. Conserv.* **34**, 151–164 (2016).
8. Reid, R. S. *et al. Fragmentation in Semi-Arid and Arid Landscapes: Consequences for Human and Natural Systems Ch. 9* (Springer, Netherlands, 2008).
9. Talbot, L. M. & Talbot, M. H. The Wildebeest in Western Masailand, East Africa. *Wildlife Monogr.* 3–88 (1963).

10. Veldhuis, M. P. *et al.* Cross-boundary human impacts compromise the Serengeti-Mara ecosystem. *Science* **363**, 1424–1428 (2019).

11. Kauffman, M. J. *et al.* Mapping out a future for ungulate migrations. *Science* **372**, 566–569 (2021).

12. Spinage, C. A. The decline of the Kalahari wildebeest. *Oryx* **26**, 147–150 (1992).

13. Perkins, J. S. Botswana: fencing out the equity issue. Cattleposts and cattle ranching in the Kalahari Desert. *J. Arid Environ.* **33**, 503–517 (1996).

14. Keene-Young, R. A thin line: Botswana's cattle fences. *Africa Environment and Wildlife* (1999).
