# Supplementary material for: Introgression and disruption of migration routes have shaped the genetic integrity of wildebeest populations
Source: Nat Commun. 2024 Apr 12;15:2921. doi: 10.1038/s41467-024-47015-y (PMC11014984; doi:10.1038/s41467-024-47015-y)
Supplement: Supplementary file 3 — Description of Additional Supplementary Files [file 41467_2024_47015_MOESM3_ESM.pdf]

Description of Additional Supplementary Files

File Name: Supplementary Data 1

Description: Summary of sample information.

File Name: Supplementary Data 2

Description: Summary of site filters for reference genomes of the blue wildebeest and domestic goat.

File Name: Supplementary Data 3

Description: Overview of genotype datasets.

File Name: Supplementary Data 4

Description: Summary of dataset and samples used in different population genetic analyses.

File Name: Supplementary Data 5

Description: Summary of the statistically non-rejected admixture graphs allowing for up to 5 migration events in qpGraph.

File Name: Supplementary Data 6

Description: Confidence intervals for demographic parameters in the fastsimcoal2 model.
